# Circular RNA hsa_circ_0004872 inhibits gastric cancer progression via the miR-224/Smad4/ADAR1 successive regulatory circuit

**DOI:** 10.1186/s12943-020-01268-5

**Published:** 2020-11-10

**Authors:** Cunying Ma, Xiaoying Wang, Fenghua Yang, Yichen Zang, Jiansong Liu, Xinyi Wang, Xia Xu, Wenjuan Li, Jihui Jia, Zhifang Liu

**Affiliations:** 1 Department of Biochemistry and Molecular Biology, Key Laboratory for Experimental Teratology of Chinese Ministry of Education, School of Basic Medical Sciences, Cheeloo College of Medicine, Shandong University, Jinan, 250012 People’s Republic of China; 2 Department of Microbiology, Key Laboratory for Experimental Teratology of Chinese Ministry of Education, School of Basic Medical Sciences, Cheeloo College of Medicine, Shandong University, Jinan, People’s Republic of China

**Keywords:** Gastric cancer (GC), hsa_circ_0004872, miR-224, Smad4, p21, ADAR1

## Abstract

**Background:**

Emerging evidence has shown that circular RNAs (circRNAs) play a crucial regulatory role in the occurrence and development of cancer. Exploring the roles and mechanisms of circRNAs in tumorigenesis and progression may help to identify new diagnostic markers and therapeutic targets. In the present study, we investigated the role and regulatory mechanism of hsa_circ_0004872 in gastric cancer (GC).

**Methods:**

qRT-PCR was used to determine the expression of hsa_circ_0004872 in GC tissues and cells. EdU, CCK-8, transwell and scratch wound healing assays were used to assess the role of hsa_circ_0004872 in GC cell proliferation, invasion and migration, respectively. Subcutaneous and tail vein tumor injections in nude mice were used to assess the role of hsa_circ_0004872 in vivo. RIP assay, biotin-coupled probe pull-down assay, FISH and luciferase reporter assay were performed to confirm the relationship between hsa_circ_0004872 and the identified miRNA. ChIP assay, luciferase reporter assay and western blot were used to determine the direct binding of Smad4 to the promoter of the ADAR1 gene.

**Results:**

In this study, we found that hsa_circ_0004872 was dramatically downregulated in GC tissues compared with adjacent noncancerous tissues. The expression level of hsa_circ_0004872 was associated with tumor size and local lymph node metastasis. Enforced expression of hsa_circ_0004872 inhibited the proliferation, invasion and migration of GC cells, whereas knockdown of hsa_circ_0004872 had the opposite effects. Nude mice experiments showed that ectopic expression of hsa_circ_0004872 dramatically inhibited tumor growth and metastasis in vivo. Moreover, we demonstrated that hsa_circ_0004872 acted as a “molecular sponge” for miR-224 to upregulate the expression of the miR-224 downstream targets p21 and Smad4. Importantly, we found that the RNA-editing enzyme ADAR1 inhibited hsa_circ_0004872 expression and further led to the upregulation of miR-224. Smad4, the downstream target of miR-224, could further affect hsa_circ_0004872 levels by directly binding to the promoter region of ADAR1 to inhibit ADAR1 expression.

**Conclusions:**

Our findings showed that hsa_circ_0004872 acted as a tumor suppressor in GC by forming a negative regulatory loop consisting of hsa_circ_0004872/miR-224/Smad4/ADAR1. Thus, hsa_circ_0004872 may serve as a potential biomarker and therapeutic target for GC.

**Supplementary information:**

**Supplementary information** accompanies this paper at 10.1186/s12943-020-01268-5.

## Introduction

Gastric cancer (GC) is one of the most common malignancies in the world and ranks third in terms of cancer-related deaths [1]. Despite improvements in clinical diagnosis and therapeutic strategies, the prognosis of GC patients remains poor due to a high recurrence rate and distant metastasis [2, 3]. Therefore, understanding the molecular mechanism of GC progression and identifying effective molecular diagnostic markers and therapeutic targets are extremely important to improve the survival rate of GC patients.

Circular RNA (circRNA) is a class of single-stranded RNAs with development/ tissue-specific expression patterns in eukaryotic cells [4]. CircRNAs are generated from the ‘back-splicing’ of precursor mRNA (pre-mRNA) transcripts to form a covalently closed circular structure without 5′ to 3′ polarity or 3′ polyA tail [4]. The unique circular structure of circRNAs confers inherent resistance to degradation by exonuclease and makes them more stable than their linear parental genes [4–6]. In addition, the expression of circRNA is evolutionarily conserved, and the expression levels of some circRNAs are much higher than those of their parental genes [7]. Recently, evidence has shown that circRNAs are aberrantly expressed in tumor tissues and cells and play indispensable roles during the occurrence and development of various tumors [8, 9], such as hepatocellular carcinoma (HCC) [10, 11], lung cancer [12], GC [13], breast cancer [14], bladder cancer [15], and glioblastoma [16]. Therefore, circRNAs have great potential as valuable diagnostic biomarkers or therapeutic targets in tumors [17]. The function of circRNAs is usually associated with their source and location in the cells. Most circRNAs are derived from the exon(s) of genes, called exonic circRNAs (ecRNAs), which are located in the cytoplasm [18] and usually exert their biological functions at the posttranscriptional level by acting as miRNA “sponges” [19] or binding to RNA-binding proteins [20, 21]. The other two kinds of circRNAs, intron circRNAs (ciRNAs) and exon-intron circular RNAs (EIciRNAs), are mostly located in the nucleus and play a regulatory role in the transcription of their parental genes [22, 23] or act as templates for protein translation [24–26].

In this study, we used next-generation sequencing of circRNAs to screen the differentially expressed circRNAs in GC and adjacent nontumor tissues and identified the downregulated hsa_circ_0004872 (also known as circMAPK1 [27, 28]), which is derived from the exons of the MAPK1 gene. Then, we investigated the role and regulatory mechanism of hsa_circ_0004872 in GC. We found that hsa_circ_0004872 inhibited GC growth, invasion and metastasis both in vitro and in vivo by acting as a “sponge” of miR-224 to upregulate p21 and Smad4 expression. Importantly, we verified that Smad4 could further regulate hsa_circ_0004872 expression by directly binding to the promoter region of the RNA-editing enzyme ADAR1 and inhibiting ADAR1 expression in GC. Therefore, hsa_circ_0004872 exerted its functions via the miR-224/Smad4/ADAR1/hsa_circ_0004872 negative regulatory circuit. This is the first report on the expression, role and regulatory mechanism of hsa_circ_0004872 in GC. Our findings indicate that hsa_circ_0004872 may act as a promising diagnostic and prognostic marker for GC patients.

## Materials and methods

### Patient samples and ethical approval

We collected 76 pairs of GC tissues and corresponding adjacent noncancerous tissues from Shandong Cancer Hospital in 2012–2013 and Taian City Central Hospital in 2016–2017. None of the patients enrolled in the study received chemotherapy or radiotherapy before surgery, and there was no evidence of any other malignancies. For each tumor, age, sex, tumor size, invasiveness and regional and distant metastases were evaluated and described in supplementary Table S1. The samples were excised from patients, immediately frozen in liquid nitrogen and stored until use. All samples were collected for research use only. The research was approved by the ethics committee of the School of Basic Medical Sciences, Shandong University.

### RNA sequencing analysis

The total RNA was isolated from the GC and adjacent nontumor tissue using TRIzol reagent (Invitrogen, USA) according to the manufacturer’s instructions. The RNA purity and concentration were determined with NanoDrop ND-1000 (NanoDrop Thermo). The RNA integrity of the samples was assesed by denaturing agarose gel electrophoresis.The Ribo-Zero rRNA Removal Kit (Illumina, San Diego, CA, USA) was used to remove the rRNA. The high-throughput whole transcriptome sequencing and subsequent bioinformatics analysis were performed by Cloud-Seq Biotech (Shanghai, China) as previously reported [29]. Paired-end reads were acquired from the Illumina HiSeq 4000 sequencer. Edger software (v3.16.5) was used to identify the differentially expressed circRNAs.

### Cell culture and transfection

The human GC cell lines AGS, BGC-823, SGC-7901 and gastric epithelial immortalized GES-1 cells were purchased from the Cell Resource Center, Institute of Biochemistry and Cell Biology at the Chinese Academy of Science (Shanghai, China). All cells were cultured in RPMI-1640 medium (GIBCO Thermo Fisher Scientific, USA) with 10% fetal bovine serum (Biological Industries, USA) and 100 U/mL penicillin-streptomycin (Life Technologies, USA). All cells were cultured at 37 °C with 5% CO_2_.

Plasmids were transfected into GC cells using X-tremeGENE HP Transfection Reagent (Roche Applied Science, Germany). SiRNAs, miRNA mimics or inhibitors were transfected into cells using Lipofectamine 2000 (Invitrogen, USA). The experiment was performed according to the manufacturer’s instructions. To construct the hsa_circ_0004872 stably overexpressing cell lines, we transfected the control vector pLCDH-circ or the hsa_circ_0004872 overexpression vector pLCDH-hsa_circ_0004872 into BGC-823 cells and then selected them with puromycin (Sigma, USA) for 2–3 weeks until hsa_circ_0004872 was stably overexpressed in the cells.

### miRNAs, siRNAs and plasmid construction

The hsa_circ_0004872 overexpression vector pLCDH-hsa_circ_0004872 was constructed by BioSune (Shanghai, China) by inserting the sequence of hsa_circ_0004872 into the pLCDH-circ expression vector. The mutated vector was constructed by changing the binding site of miR-224 in hsa_circ_0004872“GTGACTT” into “ACAGTCC” and inserting the mutated sequence into the pLCDH-circ expression vector. SiRNAs targeting hsa_circ_0004872 and the control siRNA were synthesized by Genepharma (Shanghai, China). All the siRNA sequences are listed in Table S2. Mimics and inhibitor of miR-224 were synthesized by RiboBio (Guangzhou, China). The pMIRGLO-hsa_circ_0004872 luciferase reporter plasmid was constructed by inserting the sequence of hsa_circ_0004872 into pMIRGLO (Promega, USA) between the *Xho I* and *Sal I* sites. The pMIR-p21 and pMIR-Smad4 luciferase reporter plasmids were constructed by inserting the 3’UTR fragment of p21 or Smad4 into the pMIR reporter vector (Promega, USA) between the *Spe I* and *Hind III* sites. The miR-224 complementary sequence “GTGACTT” in hsa_circ_0004872 and the 3′UTRs of p21 and Smad4 were mutated to remove the complementarity. The pGL3-ADAR1 luciferase reporter plasmid was constructed by inserting the promoter sequence of ADAR1 (+ 12 to − 1999) into the pGL3-basic vector (Promega, USA) between the *Mlu I* and *Hind III* sites. All of the primer sequences are listed in Table S3. The p-3 × flag-CMV-Smad4 plasmid and control plasmid (p-3 × flag-CMV) were kindly provided by Assistant researcher Lidong Zu (Center of Pathology, School of Medicine, Shanghai Jiao Tong University, China). pmGFP-ADAR1-p150 (Plasmid #117927) and pmGFP-ADAR1-p110 (Plasmid #117928) were purchased from Addgene (Cambridge, USA). All of the constructs were verified by sequencing.

### RNA extraction, qRT-PCR, RNase R treatment and actinomycin D treatment

The total RNA was isolated from tissues and cells using TRIzol reagent (Invitrogen, USA) according to the manufacturer’s protocol. cDNA was synthesized with random primers or miRNA-specific stem-loop primers using a Revert Aid First Strand cDNA Synthesis kit (Thermo Scientific, USA). qRT-PCR was performed on the Bio-Rad CFX96TM Real-Time PCR System (Bio-Rad). Primers for mRNAs were synthesized by BioSune (Shanghai, China), and miRNA primers were synthesized by RiboBio (Guangzhou, China). The relative RNA expression levels were analyzed using the 2^-△△Ct^ method. β_2_-M and U6 were used as the internal control genes for mRNA and miRNA, respectively. For the RNase R treatment, 2 μg total RNA was digested at 37 °C for 20 min and 70 °C for 5 min with 3 U/μg RNase R (Epicentre Technologies, USA). Then, the expression levels of linear mRNA and circular RNA were determined by qRT-PCR, RT-PCR and Northern blot. For actinomycin D (ActD) treatment, 2 μg/mLActD (Sigma-Aldrich, USA) for BGC-823 cells and 1 μg/mL ActD for SGC-7901 cells were used. Cells were collected in a series of time intervals, and the expression of linear mRNA and circRNA was detected by qRT-PCR.

### Western blot

Total proteins from GC cells were extracted with RIPA lysis buffer containing proteinase inhibitor. The protein concentrations were measured by the BCA reagent kit (Beyotime, China). The proteins were separated by SDS-PAGE and transferred to polyvinylidene difluoride membranes (Merck Millipore, Germany), which were blocked in 5% nonfat milk and then incubated with primary antibodies against p21 (1:1000, Proteintech, 10355-1-AP, China), Smad4 (1:2500, Abcam, ab40759, USA), HOXD10 (1:5000, Abcam, ab138508, USA), PTEN (1:1000, Cell Signaling Technology, #9559, USA), ADAR1 (1:4000, Abcam, ab126745, USA), myc (1:2000, Proteintech, 16286-1-AP, China), β-actin (1:4000, Cell Signaling Technology, #12262, USA) and GAPDH (1:3000, Proteintech, 10494-1-AP, China) at 4 °C overnight. Then, the membranes were washed in Tris-buffered saline with Tween, incubated with anti-mouse or anti-rabbit horseradish peroxidase-conjugated secondary antibody for 1 h at room temperature, and developed with the enhanced chemiluminescence method (Millipore, USA). β-Actin or GAPDH served as a loading control.

### Immunohistochemistry (IHC)

IHC was performed by using the kit PV-9000 (ZSGB-BIO, China) according to the manufacturer’s protocol. Paraffin sections were incubated with primary antibodies against p21 (1:300, Proteintech, 10355-1-AP, China) or Smad4 (1:100, Abcam, ab40759, USA) at 4 °C overnight. After washing in PBS, the sections were incubated with anti-mouse or rabbit horseradish peroxidase-conjugated secondary antibodies for 1 h at room temperature. Then paraffin sections were stained with DAB and hematoxylin. Finally, the paraffin sections were covered with coverslips for microscopic (Nikon, Japan) observation.

### RNA fluorescence in situ hybridization (FISH)

The Cy3-labeled hsa_circ_0004872 probes and FITC-labeled hsa-miR-224 probes were designed and synthesized by Geneseed Biotechnology Co., Ltd. (Guangzhou, China).The sequences of the probes are listed in Table S4. BGC-823 cells were grown on round coverslips, fixed, permeabilized in PBS with 0.5% Triton X-100 and dehydrated in ethanol. FISH probes were diluted (1:50), denatured, balanced and added to cells at 37 °C overnight. After hybridization, cells were stained with DAPI-Antifade for 10 min at room temperature, and the slides were sealed with rubber cement and placed in the dark for more than 20 min. Finally, the results were observed with a TCS SP2 AOBS laser confocal microscope (Leica Microsystems, Germany).

### RNA-binding protein immunoprecipitation (RIP) assay

The RIP assay was performed using the EZ-Magna RIP RNA-binding protein immunoprecipitation kit 17-701 (Merck Millipore, Germany) according to the manufacturer’s instructions. Briefly, BGC-823 cells were collected and lysed in RIP lysis buffer with protease and RNase inhibitors. The cell lysates were incubated with magnetic beads conjugated with Ago2 antibodies or IgG (Millipore) at 4 °C overnight. Then, the beads were washed and incubated with proteinase K to remove proteins. Finally, RNA was extracted and subjected to RT-PCR and agarose gel electrophoresis analysis.

### Biotin-coupled probe pull-down assay

The biotin-labeled hsa_circ_0004872 probe and the negative control probe were synthesized by RiboBio Biotech (Guangzhou, China), and the sequences are listed in Table S4. The circRIP assay was performed as previously described [10] with minor modifications. Briefly, hsa_circ_0004872-overexpressing BGC-823 cells were fixed with 1% formaldehyde for 10 min at 37 °C, and then the cells were lysed and sonicated. After centrifugation, 50 μL of the supernatant was used as input, and the remaining part was incubated with the biotin-labeled probe against hsa_circ_0004872 or negative control for 4 h at 25 °C. Then, hsa_circ_0004872 miRNAs complexes were captured with streptavidin-coupled Dynabeads (Invitrogen) overnight. On the next day, the hsa_circ_0004872/miRNAs/bead complexes were incubated with RIP wash buffer containing proteinase K. Finally, TRIzol was added to the mixture for RNA extraction and detection.

### Northern blot

The probe which spanned the back-splice junction of hsa_circ_0004872 was labeled with Digoxin. The probe sequences were listed in Table S4. Twenty micrograms total RNA was denatured in formaldehyde and then electrophoresed in a 1% agarose-formaldehyde gel. Then the RNAs were transferred onto a Hybond-N^+^ nylon membrane (Millipore).The membranes were crosslinked, pre-hybridized and hybridized with DIG-labeled DNA probes overnight. After washing, the membranes was incubated with alkaline phosphatase (AP)-conjugated anti-DIG antibodies (Roche). Immunoreactive bands were visualized after adding the chemiluminescent substrate CSPD (Roche) followed by exposure in the exposure apparatus (Tanon-520, Tianneng, China).

### Luciferase reporter assay

BGC-823 and SGC-7901 cells were seeded in 24-well plates (3 × 10^4^ cells/well). Negative control mimics or miR-224 mimics were cotransfected with the reporter plasmid into GC cells using Lipofectamine 2000. After 48 h, the cells were collected and lysed with passive lysis buffer. The luciferase activities were assessed using the Dual Luciferase Assay Kit (Promega) according to the manufacturer’s protocol. Firefly luciferase activity was normalized to Renilla luciferase activity.

### Chromatin immunoprecipitation (ChIP) assay

Treated GC cells were cross-linked in 1% (vol/vol) formaldehyde-containing medium for 10 min at 37 °C and then lysed and sonicated to disrupt chromatin DNA into fragments between 200 and 1000 bp. ChIP was performed using the ChIP Assay 17–295 kit (Merck Millipore, Germany) according to the manufacturer’s protocol. An antibody against Flag (Sigma-Aldrich, USA) was used to immunoprecipitate the DNA fragments. The protein-DNA complexes were collected with protein A Sepharose beads, eluted and reverse cross-linked. The samples were extracted with Dr.GenTLE™ Precipitation Carrier (TaKaRa Biotechnology Co., Ltd., Dalian, China). The recovered DNA was resuspended in DDW and used as the template to amplify the ADAR1 promoter. The primer sequences are listed in Table S3.

### Cell proliferation assay

Cell proliferation was detected with EdU and CCK-8 assays. The EdU assay was performed according to the protocol of the Cell-Light™ EdU Apollo®567 In Vitro Imaging Kit (RiboBio, Guangzhou, China). Briefly, the treated cells were seeded in 96-well plates and incubated with 50 μM EdU for 2 h at 37 °C. After being fixed with 4% paraformaldehyde, the cells were exposed to 100 μL of 1 × Apollo® reaction cocktail and then incubated with 5 μg/mL Hoechst 33342 to stain cell nuclei. Images were captured using a fluorescence microscope (Olympus, Tokyo, Japan). The percentage of EdU-positive cells was defined as the proliferation rate. For the CCK-8 assay, the treated cells were seeded in 96-well plates and incubated with 100 μL 10% CCK-8 solution for 4 h at 37 °C. The absorbance was measured at 450 nm with an Infinite M200 spectrophotometer (Tecan). All of the experiments were repeated three times in triplicate.

### Scratch wound healing assay

GC cells with different transfections were cultured in six-well plates at 37 °C. Scratch wounds were created by using the fine end of 10-μL pipette tips. Images of migrated cells were captured under phase-contrast microscopy at different times.

### Invasion and migration assays

The invasion and migration assays were performed with a Transwell chamber coated with Matrigel (BD Biosciences, USA) (for the invasion assay) or without Matrigel (for the migration assay). The transfected GC cells were collected and resuspended in serum-free RPMI-1640 medium. A total of 5 × 10^4^ cells for the migration assay or 1 × 10^5^ cells for the invasion assay were seeded into the upper compartment of a 24-well chamber with an 8.0 μm pore (Corning, USA). RPMI-1640 medium containing 20% FBS was added to the lower chambers as a chemoattractant. The cells were incubated for 48 h for the invasion assay or 24 h for the migration assay. Then, the cells on the upper surface of the polycarbonate membrane were removed with cotton swabs, while the cells on the lower side were fixed with 100% methanol, stained with 0.05% crystal violet and imaged under a microscope. The number of migrating cells was counted from three independent experiments.

### Xenograft nude mouse model

Five-week-old female BALB/c nude mice were purchased from the Nanjing Biomedical Research Institute of Nanjing University (Nanjing, China). For the tumor formation experiment, BGC-823 cells (4 × 10^5^) that were stably transfected with the hsa_circ_0004872 overexpression vector (pLCDH-hsa_circ_0004872) or control vector (pLCDH_circ) were subcutaneously injected into either side of the back of each mouse. Tumor size was monitored by measuring the length (L) and width (W) of the tumor every 3 days with a caliper, and the tumor volume (V) was calculated with the formula V = 1/2 × L × W^2^_._ Twenty days after injection, the mice were euthanized, and the tumors were weighed.

To investigate the effect of hsa_circ_0004872 on the metastatic ability of GC cells in nude mice, GC cells (6 × 10^5^) stably transfected with pLCDH-hsa_circ_0004872 or pLCDH_circ were injected into the tail veins of nude mice. After six weeks, the mice were sacrificed. The lungs of the nude mice were collected and stained with hematoxylin-eosin staining. All mice experiments were approved by the ethics committee of the School of Basic Medical Sciences, Shandong University and were performed according to the guidance of animal experiments in the Laboratory Animal Center of Shandong University.

### Bioinformatics analysis

hsa_circ_0004872 sequence data were obtained from circBase (http://www.circbase.org/). The target miRNAs of hsa_circ_0004872 were predicted with circular RNA interactome (https://circinteractome.nia.nih.gov), BIOINF (http://www.bioinf.com.cn/), and starBase (http://starbase.sysu.edu.cn/index.php). The binding sites of Smad4 in the ADAR1 promoter region were predicted with Jaspar (http://jaspar.genereg.net/).

### Statistical analysis

Comparisons between different groups were analyzed with GraphPad Prism v7.0 software (GraphPad Software, La Jolla, CA, USA) using a paired or unpaired t-test. Linear regression analysis was used to analyze the correlation between hsa_circ_0004872 and miR-224 expression and between Smad4 and ADAR1 expression in GC samples. Overall survival comparison between the high ADAR1 group and the low Smad4 group was conducted by log-rank (Mantel–Cox) test in the Kaplan–Meier plots. *p* < 0.05 was considered statistically significant.

## Results

### hsa_circ_0004872 is downregulated in GC tissues

**Fig. 1:**
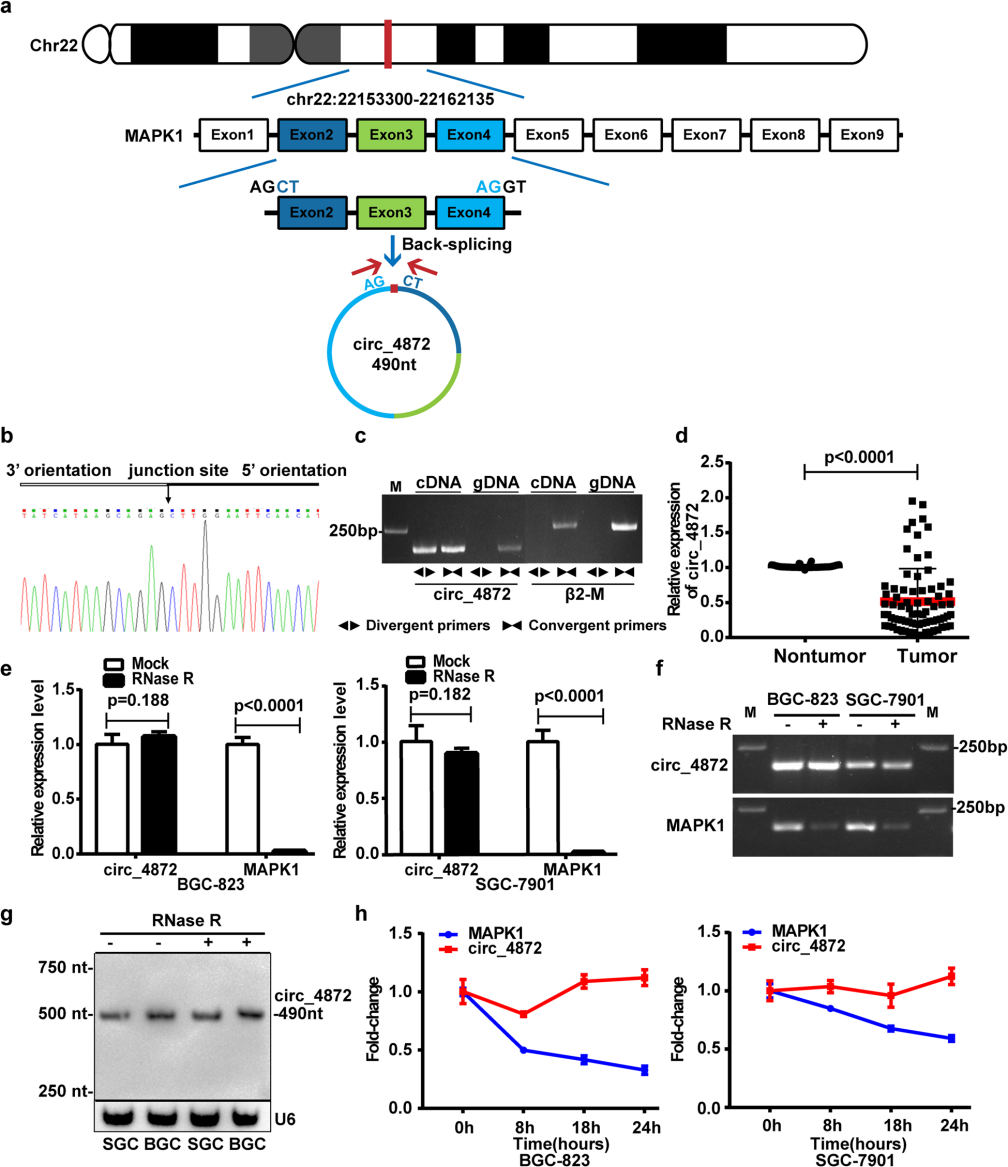
The validation, expression and stability analysis of hsa_circ_0004872 in GC. **a** Schematic diagram exhibits the formation of hsa_circ_0004872 (circ_4872) via the circularization of the exons 2, 3 and 4 from MAPK1 in Chr22 (red arrow). **b** Sanger sequencing of the RT-PCR products of hsa_circ_0004872. The black arrow indicated the splicing site of hsa_circ_0004872. **c** cDNA and gDNA of BGC-823 cells were used as the templates to amplify hsa_circ_0004872 and β_2_-M with divergent primers and convergent primers, respectively. The results showed that hsa_circ_0004872 was amplified by divergent primers in cDNA but not gDNA. The nagative control β2-M can not be amplified by divergent primers in both cDNA and gDNA. **d** qRT-PCR analysis of hsa_circ_0004872 expression in GC tissues and corresponding nontumor tissues (*n* = 76, *p* < 0.0001, Student’s t-tests). **e** and **f** The RNA levels of hsa_circ_0004872 and MAPK1 in both BGC-823 and SGC-7901 cells were detected by qRT-PCR (**e**) or RT-PCR (**f**) in the presence or absence of RNase R. **g** Northern blot analysis of the RNA level of hsa_circ_0004872 in the GC cells treated with RNase R. **h** qRT-PCR analysis of the hsa_circ_0004872 and MAPK1 expression in the GC cells under the treatment with actinomycin D. All datas were the means ± SD

To explore circRNAs expression profile in GC, we performed circRNA-seq analysis of GC tissue and corresponding nontumor tissue from Shanghai Cloud-seq Biotech Co.Ltd. (Shanghai, China).The differentially expressed circRNAs were listed in Table S5. Then, we chose the most differentially expressed circRNAs, hsa_circ_0004872, hsa_circ_0002483, hsa_circ_0000847 and hsa_circ_0001566, to be further detected with qRT-PCR in 42 paired GC and adjacent nontumor tissues. We found that hsa_circ_0004872 showed the most significant downregulation (36/42 × 100% = 85.7%, *p* < 0.0001) in GC tissues (Fig. S1). Therefore, we chose hsa_circ_0004872 as our candidate for further experiments.

hsa_circ_0004872 contains 490 nucleotides and is derived from the exon 2, 3 and 4 of human MAPK1 in chr22 (22153300-22162135) (Fig. 1a). It is also known as circMAPK1 [27, 28]. To verify the qRT-PCR results of hsa_circ_0004872 in GC tissues, we performed Sanger sequencing on the PCR products of hsa_circ_0004872. The result confirmed the head-to-tail splicing in the PCR products with the expected size and predicted splicing site (Fig. 1b). Given that head-to-tail splicing is not only the result of reverse splicing of cDNA but also the result of genomic rearrangements, we designed convergent primers and divergent primers for hsa_circ_0004872 to perform PCR using cDNA or genomic DNA from BGC-823 cells as the template. The results indicated that hsa_circ_0004872 was amplified by the divergent primers only from cDNA but not from gDNA (Fig. 1c). We used the divergent primers to further detect the expression of hsa_circ_0004872 in another 34 paired GC and nontumor tissues. The statistical analysis from the 34 pairs of samples and the first 42 pairs of samples showed that hsa_circ_0004872 was downregulated in 86.8% of the GC tissues (66/76 × 100% = 86.8%), and the expression of hsa_circ_0004872 was significantly decreased in GC tissues (*p* < 0.0001) (Fig. 1d). The clinical and pathological characteristics of these patients are shown in Table S1. Statistical analysis revealed that low hsa_circ_0004872 expression levels were correlated with advanced T stage and N stage and big tumor size in GC patients (Table 1). It has been reported that stability is one of the crucial characteristics of circRNAs [5–7]. To confirm the stability of hsa_circ_0004872, we used RNase R to treat the RNA extracted from BGC-823 and SGC-7901 cells. qRT-PCR and RT-PCR results showed that the levels of linear MAPK1 decreased sharply under RNase R treatment, but RNase R failed to degrade hsa_circ_0004872 (Fig. 1e and f)**.** Northern blot result also verified the stability of hsa_circ_0004872 (Fig. 1g). To further confirm the circular characteristics of hsa_circ_0004872, we used the transcription inhibitor ActD to treat BGC-823 and SGC-7901 cells for different times. As shown in Fig. 1h, the mRNA level of linear MAPK1 decreased gradually with time, while the level of hsa_circ_0004872 was more stable and resistant to ActD treatment.

**Table 1:** Correlations between the expression of hsa_circ_0004872 and various clinicopathological features in 76 GC patients

Characteristics	Cases	hsa_circ_0004872 expression		*P* value
		low	high	
All cases	76	42	34	
Age(years)				
≤ 60	36	23	13	0.172
> 60	40	19	21	
Gender				
Male	48	28	20	0.633
Female	28	14	14	
T stage				
T1-T2	16	5	11	0.046*
T3-T4	60	37	23	
N stage				
N0-N1	31	12	19	0.0201*
N2-N3	45	30	15	
M stage				
M0	66	35	31	0.4972
M1	10	7	3	
Tumor size (cm)				
≤ 5	31	11	20	0.005**
> 5	45	31	14	

(*:*p*<0.05; **:*p*<0.01)

### hsa_circ_0004872 inhibits the proliferation, invasion and migration of GC cells

**Fig. 2:**
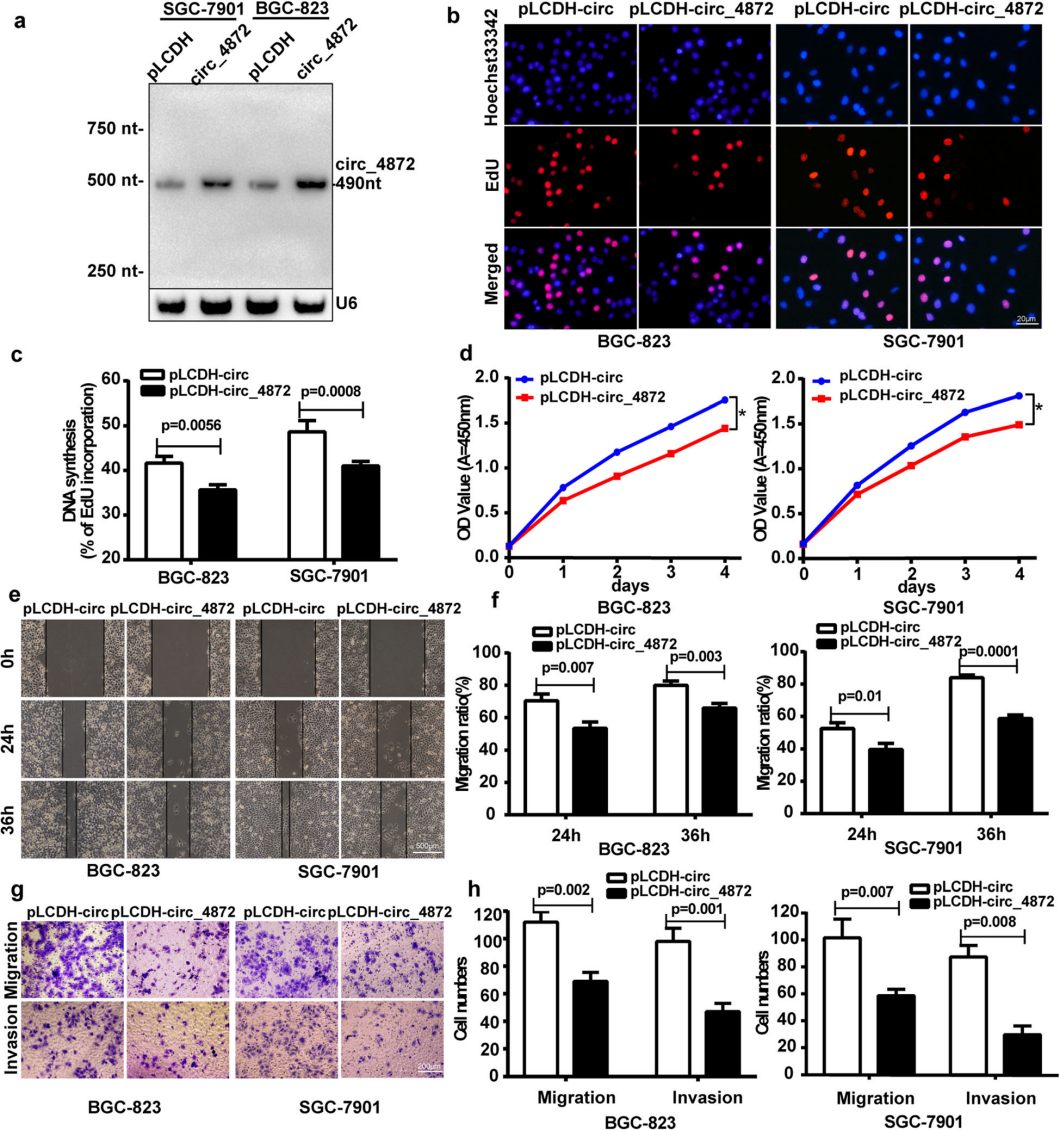
hsa_circ_0004872 overexpression inhibits GC cells proliferation, invasion and migration in vitro. **a** Northern blot was used to detect the expression of hsa_circ_0004872 in BGC-823 and SGC-7901 cells transfected with hsa_circ_0004872 overexpression vector (circ_4872) or the control vector (pLCDH). **b** EdU analysis of the cell proliferation ability in BGC-823 and SGC-7901 cells transfected with pLCDH-circ_4872 or pLCDH-circ. Representative images are shown.Scale bar: 20 μm. **c** Statistical analysis of the EdU-positive cell ratio in transfected GC cells. **d** CCK-8 analysis of the cell proliferation ability in BGC-823 and SGC-7901 cells transfected with pLCDH-circ_4872 or pLCDH-circ. **e** Scratch wound healing assays in transfected BGC-823 and SGC-7901 cells .Representative images are shown. Scale bar: 500 μm. **f** Statistical analysis of the cell migration in the scratch wound healing assays.The data are expressed as the means±SD from three experiments. **g** Transwell invasion and migration assay in transfected BGC-823 and SGC-7901 cells. Scale bar: 200 μm. **h** Statistical analysis of the cell numbers passing through the transwell filter in the transfected BGC-823 and SGC-7901 cells. The data are expressed as the means ± SD from three experiments

To investigate the biological role of hsa_circ_0004872 in GC cells, we constructed the hsa_circ_0004872 overexpression vector pLCDH-hsa_circ_0004872 and transfected the overexpression vector or the control vector (pLCDH-circ) into the GC cells BGC-823 and SGC-7901. The transfection efficiency was validated by Northern blot and qRT-PCR (Fig. 2a and Fig. S2A). Then, we used EdU and CCK-8 assays to detect the proliferation ability and used the scratch wound healing and transwell assays to detect the invasion and migration abilities of the GC cells. The results showed that enforced expression of hsa_circ_0004872 significantly suppressed both the proliferation ability (Fig. 2b-d) and invasion and migration ability of GC cells (Fig. 2e-h).

To further determine whether hsa_circ_0004872 knockdown has the opposite effects, we designed two siRNAs to specifically target the junction site of hsa_circ_0004872 (Fig. S3 A). We determined the expression level of hsa_circ_0004872 in different GC cells and found that the expression of hsa_circ_0004872 was relatively low in SGC-7901 cells (Fig. S4). Therefore, we only used BGC-823 cells to perform the knockdown experiment. We transfected the siRNAs into BGC-823 cells. The qRT-PCR results showed that the expression of hsa_circ_0004872 was significantly decreased in hsa_circ_0004872 siRNA-transfected cells, while the expression level of the parental gene MAPK1 showed no obvious changes (Fig. S3B). Functionally, the EdU assay revealed that the proliferation of BGC-823 cells increased in the hsa_circ_0004872 siRNA group compared with the control group (*p* < 0.01) (Fig. S3C and D). Moreover, the scratch wound healing assay and transwell assay showed that hsa_circ_0004872 siRNAs significantly promoted the invasion and migration ability of GC cells (Fig. S3 E-H).

### hsa_circ_0004872 acts as a “molecular sponge” for miR-224

**Fig. 3:**
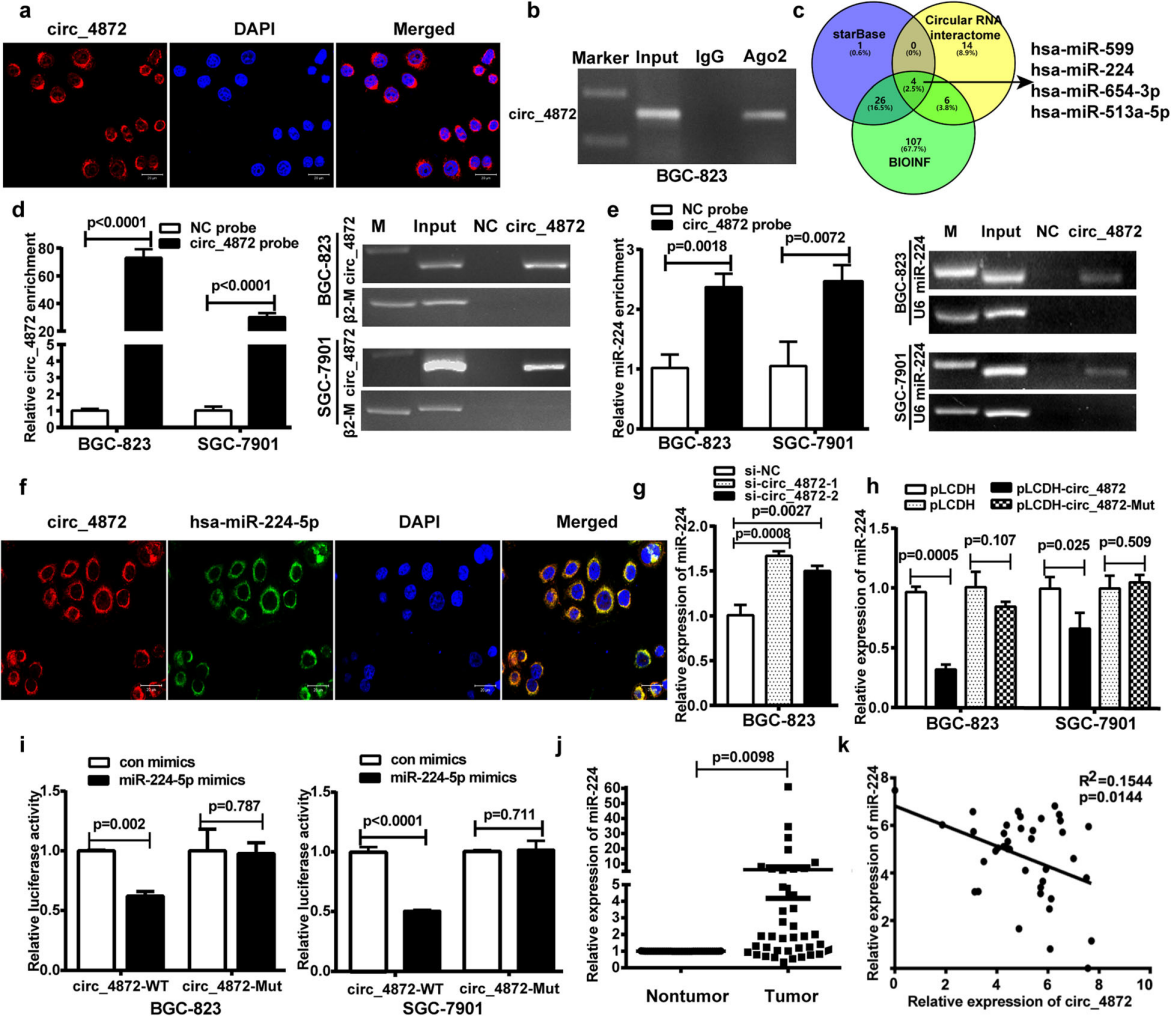
hsa_circ_0004872 acts as a “molecular sponge” for miR-224. **a** FISH experiment was used to detect the subcellular localization of hsa_circ_0004872 **(**circ_4872) in GC cells. **b** RIP analysis of hsa_circ_0004872 level in the immunoprecipitate of AGO2 antibody from GC cells. **c** Schematic diagram exhibiting the overlapping of different databases to predict the miRNAs with potential binding abilities of hsa_circ_0004872. **d** and **e** Lysates prepared from hsa_circ_0004872 overexpressing BGC-823 cells and SGC-7901 cells were incubated with biotinylated probes against hsa_circ_0004872 and then performed RNA pull-down assay. qRT-PCR and RT-PCR were used to determine the level of hsa_circ_0004872 (**d**) and miR-224 (**e**). **f** FISH analysis of the subcellular localization of hsa_circ_0004872 and miR-224 in GC cells. **g** qRT-PCR was used to determine the level of miR-224 in BGC-823 cells transfected with hsa_circ_0004872 siRNAs. **h** qRT-PCR was used to determine the level of miR-224 in BGC-823 and SGC-7901 cells transfected with hsa_circ_0004872 overexpression vector (pLCDH-circ_4872) or the mutated hsa_circ_0004872 overexpression vector (pLCDH-circ_4872-mut). **i** The wild-type (WT) or mutant (Mut) reporter constructs was cotransfected with control or miR-224 mimics into BGC-823 and SGC-7901 cells, and the dual luciferase activity was determined at 48 h after transfection. **j** qRT-PCR analysis of miR-224 expression in GC tissues and corresponding nontumor tissues with paired t-tests (*n* = 39, *p* = 0.0098). **k** Regression analysis of GC tissue showed a negative correlation between miR-224 and hsa_circ_0004872 (*n* = 39). All datas were the means ± SD

Since the functions of circRNAs are usually related to their different localization in cells [15], we detected the subcellular localization of hsa_circ_0004872 in GC cells via the FISH assay. The results indicated that hsa_circ_0004872 mainly existed in the cytoplasm of GC cells (Fig. 3a). It has been reported that circRNAs in the cytoplasm usually act as miRNA “molecular sponges” [19]. Therefore, we performed a RIP assay with an AGO2 antibody to explore whether hsa_circ_0004872 could bind to miRNAs in GC cells. As shown in Fig. 3b, hsa_circ_0004872 could be amplified from the immunoprecipitate pulled down by the AGO2 antibody, suggesting that hsa_circ_0004872 binds with miRNAs via AGO2.

Then, we used different databases (circular RNA interactome, BIOINF and starBase) to predict the potential miRNAs that bind with hsa_circ_0004872 (Table S6) and found 4 miRNAs (hsa-miR-224, hsa-miR-654-3p, hsa-miR-513a-5p and hsa-miR-599) from the overlap of the three databases (Fig. 3c). Among these miRNAs, we found that only hsa-miR-224 (miR-224) exerts oncogenic functions according to previous reports. Therefore, we performed a pull-down assay with a biotin-coupled hsa_circ_0004872 probe to determine whether hsa_circ_0004872 could interact with miR-224. The results showed that the biotin-coupled hsa_circ_0004872 probe group exhibited an obvious enrichment of miR-224 compared with the negative control group (Fig. 3d and e), suggesting that hsa_circ_0004872 could directly interact with miR-224. FISH assays showed that hsa_circ_0004872 colocalized with miR-224 in the cytoplasm of GC cells (Fig. 3f). qRT-PCR results showed that hsa_circ_0004872 knockdown increased miR-224 levels (Fig.3g) and hsa_circ_0004872 overexpression suppressed miR-224 expression, while miR-224 binding site mutated hsa-circ_0004872 overexpression has no obvious effect on the expression of miR-224 (Fig. S2B and Fig. 3h). To further determine whether miR-224 could directly target hsa_circ_0004872 in GC cells, we performed a dual luciferase assay. We constructed an hsa_circ_0004872 wild-type luciferase reporter vector (pMIRGLO-hsa_circ_ 0004872-WT) containing the binding sequences of miR-224 and a mutant luciferase reporter vector (pMIRGLO- hsa_circ_0004872-Mut) in which the binding sequences were mutated (Fig. S5A). BGC-823 and SGC-7901 cells were cotransfected with the WT or Mut luciferase reporter and miR-224 mimics. The results showed that hsa_circ_0004872 wild-type luciferase reporter activity was significantly suppressed by miR-224 mimics, while the activity of the Mut luciferase reporter was not affected by miR-224 mimics (Fig. 3i). These results suggested that miR-224 was a direct target of hsa_circ_0004872 in GC cells.

Additionally, we investigated the expression of miR-224 in paired GC and corresponding nontumor tissues and found that the expression level of miR-224 was significantly increased in GC tissues (*p* = 0.0098) (Fig. 3j and Fig. S6A). Statistical analysis showed that miR-224 expression was negatively associated with hsa_circ_0004872 in these GC samples (*p* = 0.0144) (Fig. 3k). Taken together, these results suggest that hsa_circ_0004872 functions as a “molecular sponge” for miR-224 in GC cells.

### miR-224 promotes the proliferation, invasion and migration of GC cells by targeting the 3′-UTR of p21 and Smad4

**Fig. 4:**
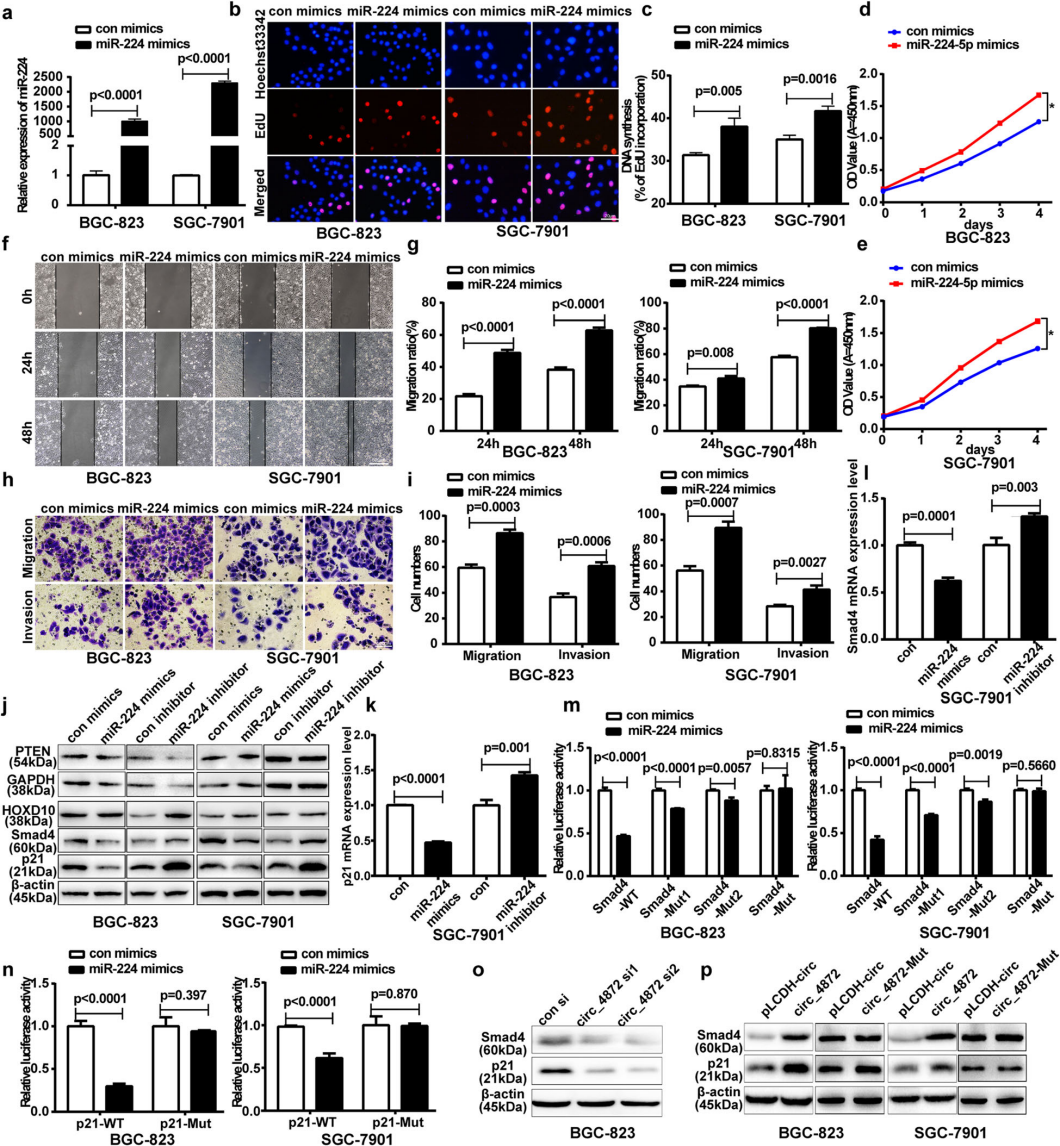
miR-224 mimics facilitates the proliferation, invasion and migration of GC cells by targeting the 3′-UTR of Smad4 and p21 directly. **a** qRT-PCR analysis of miR-224 levels in BGC-823 and SGC-7901 cells transfected with miR-224 mimics or the control mimics. **b** EdU analysis of the cell proliferation ability in BGC-823 and SGC-7901 cells transfected with miR-224 mimics or the control mimics. Scale bar: 20 μm. **c** Statistical analysis of the EdU-positive cell ratio in transfected GC cells. **d** and **e** CCK-8 analysis of the cell proliferation ability in BGC-823 (**d**) and SGC-7901 (**e**) cells transfected with miR-224 mimics or the control mimics. **f** The scratch wound healing assays in BGC-823 and SGC-7901 cells transfected with miR-224 mimics or the control mimics. Scale bar: 500 μm. **g** Statistical analysis of the cell migration in the scratch wound healing assays. **h** Transwell invasion and migration assay in BGC-823 and SGC-7901 cells transfected with miR-224 mimics or the control mimics. Scale bar: 100 μm. **i** Statistical analysis of the cell numbers passing through the transwell chamber in the transfected BGC-823 and SGC-7901 cells. All datas were the means ± SD. **j** Western blot analysis of the protein expression level of PTEN, HOXD10, p21 and Smad4 in transfected GC cells. **k** qRT-PCR analysis of the mRNA expression level of p21 in transfected GC cells. **l** qRT-PCR analysis of the mRNA expression level of Smad4 in transfected GC cells. **m** The pMIR-Smad4-WT or pMIR-Smad4-Mut reporter construct was cotransfected with control or miR-224 mimics into BGC-823 and SGC-7901 cells, and the dual luciferase activity was determined at 48 h after transfection. **n** The pMIR-p21-WT or pMIR-p21-Mut reporter construct was cotransfected with control or miR-224 mimics into BGC-823 and SGC-7901 cells, and the dual luciferase activity was determined at 48 h after transfection. All datas were the means ± SD. **o** Western blot analysis of Smad4 and p21 protein level in BGC-823 cells transfected with hsa_circ_0004872 siRNAs. **p** Western blot analysis of Smad4 and p21 protein level in GC cells transfected with hsa_circ_0004872 overexpression vector (circ_4872) or the mutated hsa_circ_0004872 overexpression vector (circ_4872-Mut)

Having confirmed the interaction of hsa_circ_0004872 and miR-224, we next sought to explore the biological role of miR-224 in gastric carcinogenesis and progression. We transfected the miR-224 mimics or inhibitor into GC cells. The transfection efficiency was verified by qRT-PCR (Fig. 4a; Fig. S7A). Then, EdU and CCK-8 assays revealed that miR-224 mimics promoted, whereas its inhibitor suppressed, GC cell proliferation (Fig. 4b-e; Fig. S7B-D). Moreover, scratch wound healing assays and transwell assays showed that miR-224 mimics promoted, whereas its inhibitor suppressed, cell invasion and migration (Fig. 4f-i; Fig. S7E-H).

Smad4 [30, 31], p21 [32], HOXD10 [33] and PTEN [34] are validated targets of miR-224 in other tumors. To determine whether miR-224 can regulate these targets in GC cells, we transfected the miR-224 mimics or inhibitor into GC cells and used western blot and qRT-PCR to determine the expression levels of these genes. As shown in Fig. 4j, miR-224 mimics significantly decreased, whereas miR-224 inhibitor increased, the protein levels of p21 and Smad4, although miR-224 mimics or inhibitor had no obvious effect on the expression levels of HOXD10 and PTEN. qRT-PCR results further showed that miR-224 mimics significantly decreased, whereas miR-224 inhibitor increased, the mRNA levels of p21 and Smad4 (Fig. 4k, l).

To determine whether miR-224 could directly target the 3’UTRs of Smad4 and p21 in GC cells, we cloned the wild-type 3′-UTR sequences and the mutant sequences of p21 and Smad4 to construct wild-type luciferase reporter vectors (pMIR-Smad4(p21)-3’UTR-WT) and mutant vectors (Fig. S5B). GC cells were cotransfected with WT or Mut luciferase reporter plasmids and miR-224 mimics. Dual luciferase reporter assay results showed that miR-224 mimics strongly reduced the wild-type luciferase reporter activity of Smad4 and p21, while the mimics had no obvious effect on the Mut luciferase reporter activity of Smad4 and p21 (Fig. 4m, n). These results suggested that Smad4 and p21 were the direct targets of miR-224 in GC cells.

We further determined the effects of hsa_circ_0004872 on Smad4 and p21 expression and found that hsa_circ_0004872 knockdown reduced the expression of Smad4 and p21 in GC cells (Fig. 4o) and the enforced expression of hsa_circ_0004872 significantly increased the p21 and Smad4 protein levels, while mutated hsa_circ_0004872 has no obvious effect on the expression of p21 and Smad4. (Fig. 4p).

### hsa_circ_0004872 exerts its antitumor effect via miR-224

**Fig. 5:**
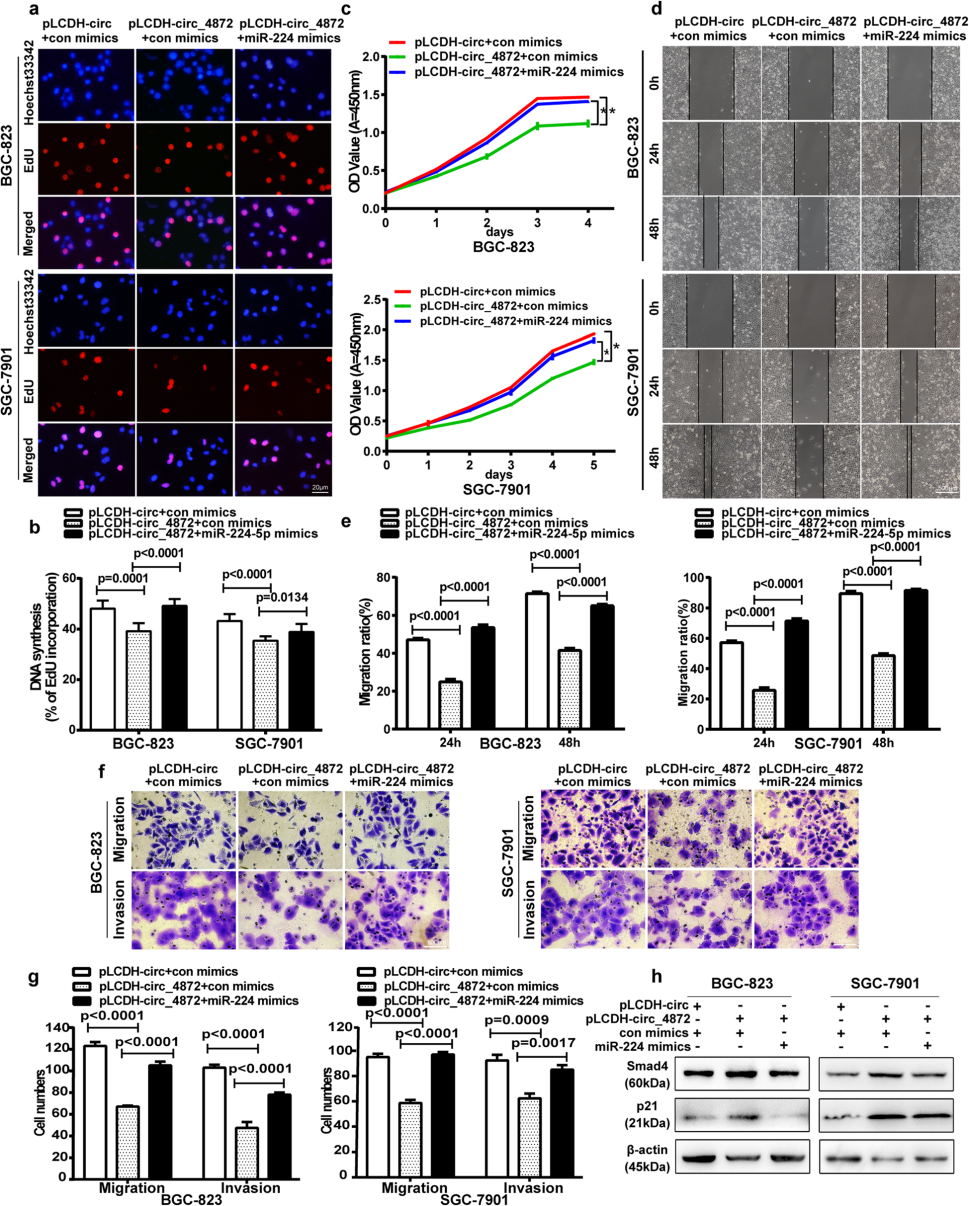
hsa_circ_0004872 regulates the proliferation, invasion and migration of GC cells via miR-224. **a** EdU analysis of the cell proliferation ability in SGC-7901 and BGC-823 cells cotransfected with pLCDH-circ+control mimics or pLCDH-hsa_circ_0004872 + control mimics or pLCDH-hsa_circ_0004872 + miR-224 mimics.Scale bar: 20 μm. **b** Statistical analysis of the EdU-positive cell ratio in the GC cells with different cotransfection. **c** CCK-8 analysis of the cell proliferation ability in cotransfected BGC-823 and SGC-7901 cells. **d** The scratch wound healing assays in BGC-823 and SGC-7901 cells with different cotransfection. Scale bar: 500 μm. **e** Statistical analysis of cell migration in the scratch wound healing assays. **f** Transwell invasion and migration assay in treated BGC-823 and SGC-7901 cells. Scale bar: 100 μm. **g** Statistical analysis of the cell numbers passing through the transwell chamber in treated BGC-823 and SGC-7901 cells. **h** Western blot analysis of the protein expression level of p21 and Smad4 in BGC-823 and SGC-7901 cells with different cotransfection. All datas were the means ± SD

To determine whether hsa_circ_0004872 functions as a tumor suppressor gene via miR-224, we performed rescue experiments. We cotransfected the hsa_circ_0004872 overexpression vector (pLCDH-circ_0004872) and miR-224 mimics into GC cells to determine whether the tumor-suppressing effect of hsa_circ_0004872 could be blocked by miR-224 mimics. The results showed that miR-224 mimics could partly attenuate the inhibition of cell proliferation (Fig. 5a-c), invasion and migration (Fig. 5d-g) mediated by hsa_circ_0004872 overexpression.

We next investigated whether hsa_circ_0004872-mediated upregulation of p21 and Smad4 can be abolished by miR-224 mimics. As shown in Fig. 5h, the upregulation of Smad4 and p21 mediated by hsa_circ_0004872 was partly rescued by miR-224 mimics (Fig. 5h). Therefore, these data demonstrated that hsa_circ_0004872 inhibited GC proliferation, invasion and migration, at least partly through miR-224.

### hsa_circ_0004872 impaired tumorigenesis and metastasis in vivo

**Fig. 6:**
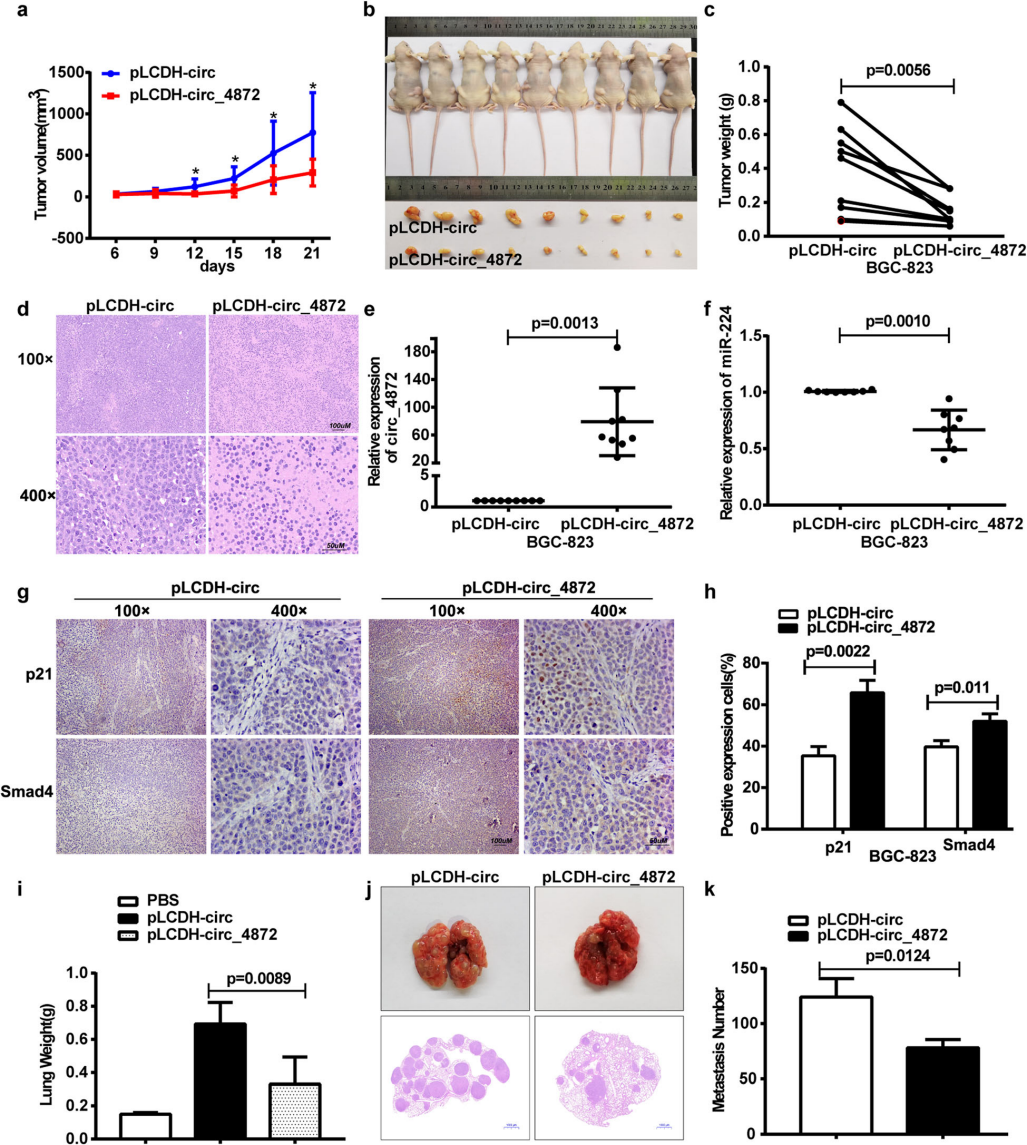
hsa_circ_0004872 serves as a sponge for miR-224 to inhibit the tumor growth and metastasis of GC in vivo. **a** The nude mice were subcutaneously injected with BGC-823 cells stably transfected with hsa_circ_0004872 (pLCDH-circ_0004872) (right) or the control BGC-823 cells (pLCDH-circ) (left) in the two flanks of the mice.After 6 days, the subcutaneous tumor size was measured every 3 days and the tumor volume was calculated. **b** The tumor-bearing mice and the dissected tumors were photographed and shown. A ruler was used to indicate the size of the tumors. **c** The tumor weight with different cells was shown. **d** Hematoxylin and eosin (HE) staining was used to detect the tumors. **e** qRT-PCR analysis of the expression of hsa_circ_0004872 in the tumors. **f** qRT-PCR analysis of the expression of miR-224 in the tumors. Data were the means ± SD. **g** IHC analysis of the expression of p21 and Smad4 in the tumors. **h** Statistical analysis of the percentage of IHC positive exprssion cells in the tumors. **i** BGC-823 cells stably transfected with hsa_circ_0004872 (pLCDH-circ_0004872) or the control BGC-823 cells (pLCDH-circ) were injected into the tail vein of the nude mice. Six weeks later, the nude mice were dissected. The mice lungs were weighed. **j** Hematoxylin and eosin (HE) staining of lung tissues was used to detect the metastasis nodules. **k** The metastatic nodules in the mice lung were counted. All datas were the means ± SD

To investigate whether hsa_circ_0004872 suppressed the tumorigenesis and metastasis of GC cells in vivo, we performed subcutaneous injection and tail vein injection in nude mice. The subcutaneous injection results showed that the mean tumor volume in the hsa_circ_0004872 overexpression group was much smaller than that in the control group (Fig. 6a, b), and the average tumor weight in the hsa_circ_0004872 overexpression group was much lighter than that in the control group (Fig. 6c). HE staining showed that all the tumors were solid tumors (Fig. 6d). qRT-PCR analysis demonstrated that the expression of hsa_circ_0004872 was elevated (Fig. 6e), whereas miR-224 expression was decreased (Fig. 6f), in the tumors from the hsa_circ_0004872 overexpression group, which was consistent with what we observed in vitro. IHC results showed that the expression of p21 and Smad4 was increased in the tumors from the hsa_circ_0004872 overexpression group (Fig. 6g,h). In the tail vein injection assay, we observed that both the mice injected with the control group and the pLCDH-hsa_circ_0004872 group formed metastatic nodules at the surface of the lungs, but the lungs from mice injected with pLCDH-hsa_circ_0004872 cells were significantly smaller and lighter than those from mice injected with pLCDH-circ cells (Fig. 6i). Additionally, mice injected with pLCDH-hsa_circ_0004872 cells had much fewer metastatic nodules in the lungs than mice injected with pLCDH-circ cells (Fig. 6j, k). Taken together, these results demonstrate that hsa_circ_0004872 efficiently inhibited GC cells growth and metastasis in vivo.

### hsa_circ_0004872 is regulated by the RNA-editing enzyme ADAR1

**Fig. 7:**
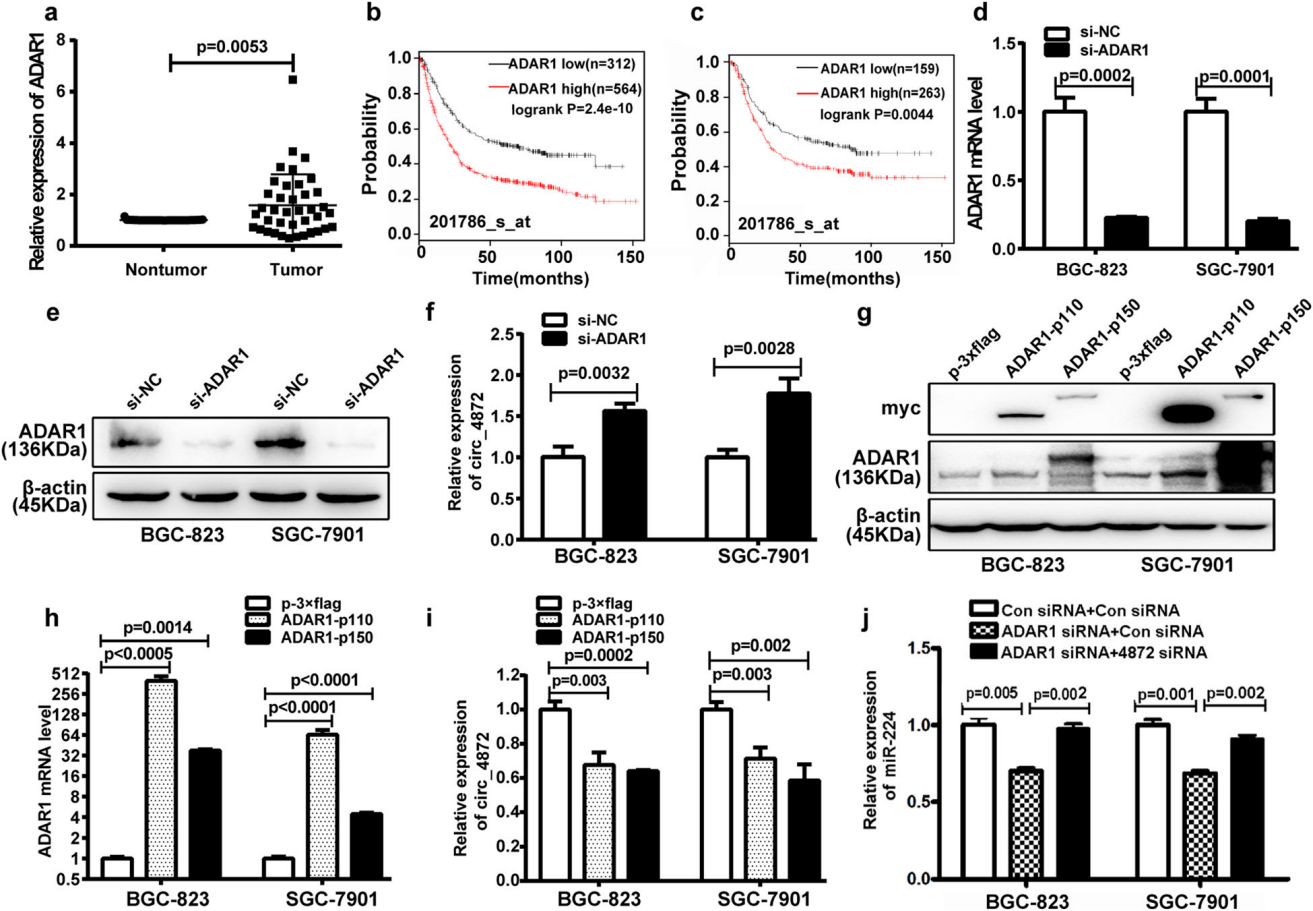
ADAR1 is up-regulated in GC tissues and regulates the expression of hsa_circ_0004872 in GC cells. **a** The expression level of ADAR1 was detected with qRT-PCR and analyzed with paired t-tests in GC tissues and the adjacent nontumor tissues (*n* = 39, *p* = 0.0053). **b** The Kaplan-Meier Plotter database (201786_s_at) analyzed the ADAR1 levels in relation to the overall survival of patients with GC. **c** The Kaplan-Meier Plotter database (201786_s_at) analyzed the ADAR1 levels in relation to the overall survival of patients with lymph node metastasis of GC. **d** qRT-PCR analysis of the mRNA expression level of ADAR1 in GC cells transfected with ADAR1 siRNA (si-ADAR1) or control siRNA (si-NC). **e** Western blot analysis of ADAR1 protein level in BGC-823 and SGC-7901 cells transfected with ADAR1 siRNA or control siRNA. **f** qRT-PCR analysis of the expression level of hsa_circ_0004872 in GC cells transfected with ADAR1 siRNA or control siRNA. **g** Western blot analysis of the expression level of ADAR1 and the label myc in BGC-823 and SGC-7901 cells transfected with ADAR1 overexpression vector (ADAR1-p110 and ADAR1-p150) or the control vector (p-3 × flag). **h** qRT-PCR analysis of the mRNA level of ADAR1 in BGC-823 and SGC-7901 cells transfected with ADAR1 overexpression vector or the control vector. **i** qRT-PCR analysis of hsa_circ_0004872 expression in BGC-823 and SGC-7901 cells transfected with ADAR1 overexpression vector or or the control vector. **j** qRT-PCR analysis of miR-224 expression in BGC-823 and SGC-7901 cells with different cotransfection. All datas were the means ± SD

We next sought to explore which factors lead to the downregulation of hsa_circ_0004872 in GC. According to previous reports, the formation of circRNA is regulated by RNA binding proteins [35], such as MBl [22], QKI [36, 37], and ADAR1 [38, 39]. We analyzed the public NCBI GEO databases GSE27342 and GSE66229 and found that only ADAR1 expression was significantly higher in GC tissues than in nontumor tissues in both GSE27342 and GSE66229 (*p* < 0.0001) (Fig. S8A-F). Our qRT-PCR results also confirmed the higher expression level of ADAR1 in GC tissues compared with the adjacent nontumor tissues (Fig. 7a, Fig. S6B), which was consistent with the results from Dou et al. [40]. Kaplan-Meier Plotter Database (201786_s_at) analysis of overall survival (Fig. 7b) and survival with lymph node metastasis (Fig. 7c) indicated that higher expression of ADAR1 in GC tissues was associated with shorter survival period. ADAR1 is an RNA editing enzyme that catalyzes the deamination of adenosine into inosine (A-to-I) and prevents the link between exon ends and circRNA formation [38, 41]. Thus, we wondered whether ADAR1 contributed to the downregulation of hsa_circ_0004872 in GC. Therefore, we transfected ADAR1 siRNA or the ADAR1 overexpressing vector (pmGFP- ADAR1-p110 or pmGFP-ADAR1-p150) into GC cells. The transfection efficiency was validated by western blot and qRT-PCR (Fig. 7d, e, g, h). Then, qRT-PCR was used to examine the expression of hsa_circ_0004872. The results showed that ADAR1 knockdown increased the expression of hsa_circ_0004872 (Fig. 7f) and that overexpression of ADAR1 led to the downregulation of hsa_circ_0004872 in GC cells (Fig. 7i). We further investigated whether ADAR1 can regulate miR-224 expression via hsa_circ_0004872. As shown in Fig. 7j, ADAR1 knockdown led to the downregulation of miR-224, and the downregulation of miR-224 mediated by ADAR1 siRNA was partly reversed by hsa_circ_0004872 siRNA. Therefore, these data demonstrated that ADAR1 regulated miR-224 expression via hsa_circ_0004872.

### Smad4 regulates hsa_circ_0004872 expression via ADAR1

**Fig. 8:**
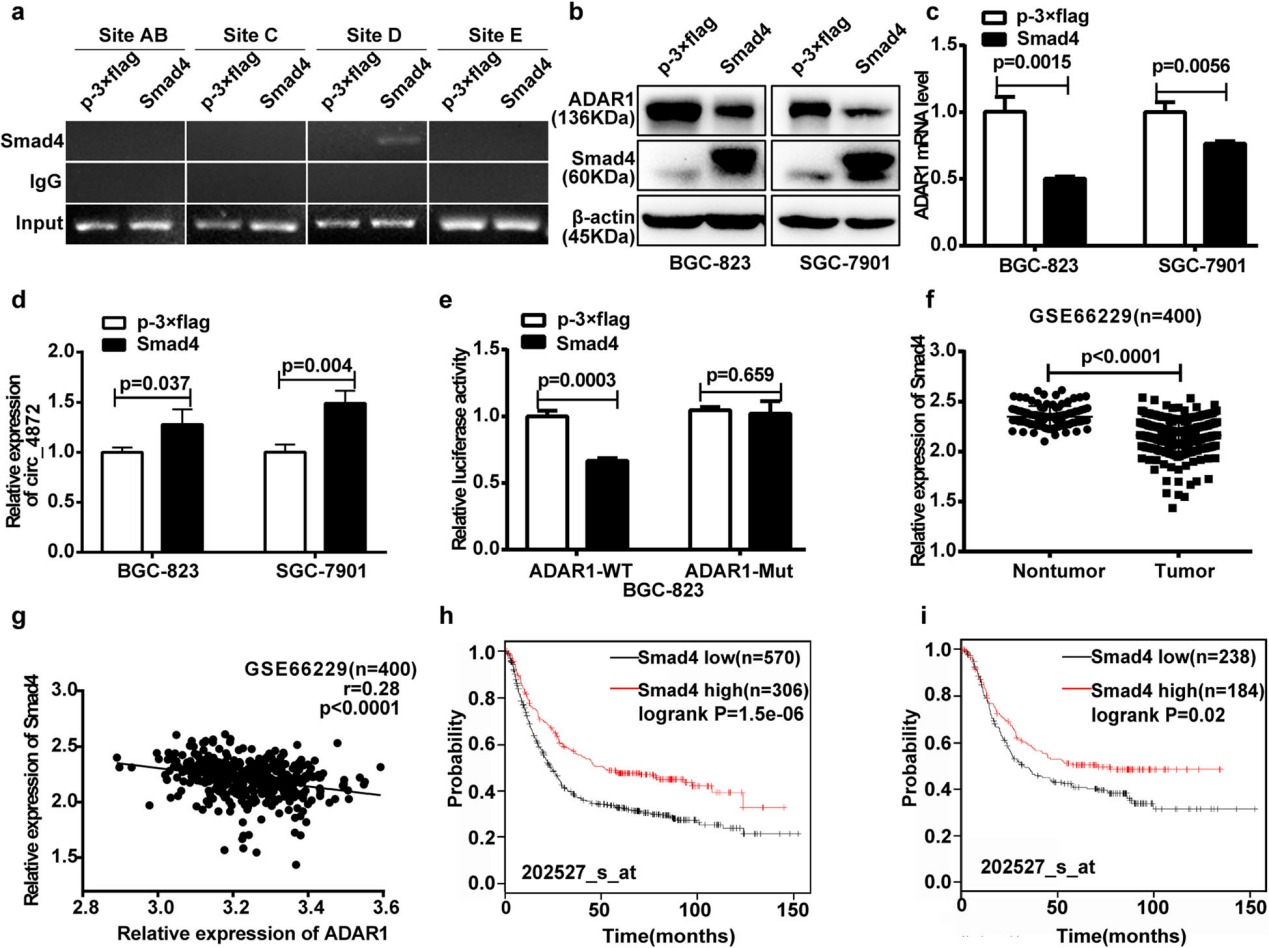
Smad4 regulates the expression of ADAR1 and hsa_circ_0004872 in GC cells. **a** BGC-823 cells transfected with p-3 × flag-CMV-Smad4 or p-3 × flag-CMV-14 were subjected to ChIP analysis. Their chromatin was immunoprecipitated with anti-flag antibody, and the ADAR1 promoter region containing the predicted Smad4 binding sites was amplified by PCR. An evident signal was found in the cells transfected with p-3 × flag-CMV-Smad4 in site D. **b** The protein expression level of ADAR1 was detected by western blot in BGC-823 and SGC-7901 cells transfected with Smad4 overexpression vector (p-3 × flag-CMV-Smad4) or the control vector (p-3 × flag- CMV-14). **c** The mRNA expression level of ADAR1 was analyzed with qRT-PCR in BGC-823 and SGC-7901 cells transfected with Smad4 overexpression vector or the control vector. **d** qRT-PCR analysis of the mRNA expression level of hsa_circ_0004872 (circ_4872) in BGC-823 and SGC-7901 cells transfected with Smad4 overexpression vector or the control vector. **e** The pGL3-ADAR1-WT or Mut reporter construct was co-transfected with the overexpression vector of Smad4 or the control vector into BGC-823 cells, and the dual luciferase activity was determined at 48 h after transfection. **f** The expression level of Smad4 was analyzed with unpaired t-tests (*n* = 400, *p* < 0.0001) in NCBI GEO (GSE66229). **g** Regression analysis of GC tissue showed a negative correlation between ADAR1 and Smad4 in NCBI GEO (GSE66229, *n* = 400). **h** The Kaplan-Meier Plotter database (202527_s_at) analyzed the correlation between Smad4 expression and the overall survival of patients with GC. **i** The Kaplan-Meier Plotter database (202527_s_at) analyzed the correlation between Smad4 expression levels and the overall survival of patients with lymph node metastasis of GC. All datas were the means ± SD

Given that hsa_circ_0004872 acts as a “molecular sponge” of miR-224 to upregulate the miR-224 target Smad4, we asked whether Smad4, as a transcription factor, can regulate ADAR1 to further lead to the dysregulation of hsa_circ_0004872. We used software to analyze the promoter of ADAR1 and found 5 potential binding sites of Smad4 at the region from nucleotide − 1993 to − 1981 (Site A), − 1959 to − 1947 (Site B), − 1590 to − 1578 (Site C), − 1280 to − 1268 (Site D) and − 760 to − 748 (Site E). Then, we performed a ChIP assay to determine the binding of Smad4 on the promoter of ADAR1. We transfected BGC-823 cells with the overexpression vector of Smad4 (p-3 × flag-CMV-Smad4) or the control vector (p-3 × flag-CMV) and used the flag antibody to precipitate the promoter fragment spanning these five sites. The results illustrated in Fig. 8a show a clear signal in Site D of the ADAR1 promoter in the cells transfected with p-3 × flag-CMV-Smad4.

To further determine whether Smad4 regulates ADAR1 and hsa_circ_0004872 expression, we transfected p-3 × flag-CMV-Smad4 or p-3 × flag-CMV-14 into GC cells. Western blot and qRT-PCR results showed that Smad4 overexpression led to a significant downregulation of the protein and mRNA levels of ADAR1 (Fig. 8b, c), while hsa_circ_0004872 was upregulated after transfection (Fig. 8d). We further constructed a luciferase reporter vector containing the fragment from − 1999 to + 12 in the ADAR1 promoter (pGL3-ADAR1-promoter-WT), which contained the sequence of Site D, and a mutant luciferase reporter vector (pGL3-ADAR1-promoter-Mut) in which the Site D sequence was mutated (Fig. S5C). BGC-823 cells were cotransfected with the WT or Mut luciferase reporter and the Smad4 overexpression vector. The luciferase reporter assay showed that Smad4 overexpression decreased the WT promoter activity of ADAR1, whereas it had no effect on the Site D Mut promoter activity of ADAR1 (Fig. 8e).

We next analyzed the expression of Smad4 in the public database NCBI GEO (GSE66229) and found that the expression of Smad4 was lower in GC tissues than in adjacent nontumor tissues (*p* < 0.0001) (Fig. 8f). Statistical analysis showed that there was a highly significant negative correlation between ADAR1 and Smad4 in these samples (*p* < 0.0001) (Fig. 8g). Kaplan-Meier Plotter Database (202527_s_at) analysis of overall survival (Fig. 8h) and survival with lymph node metastasis (Fig. 8i) indicated that lower expression of Smad4 in GC tissues resulted in a shorter survival period.

## Discussion

Circular RNA (circRNA) is a kind of single-stranded RNA that is widely found in eukaryotic cells [4]. In contrast to linear RNAs, circRNAs form a covalently closed loop structure, which makes them more stable than their linear host genes. Recently, evidence has accumulated that circRNAs play important roles in different biological processes, especially in the tumorigenesis, invasion and metastasis [9–16]. However, the function and regulatory mechanism of circRNAs in GC remain largely unknown. In this study, we focused on hsa_circ_0004872, which originates from the back-splice of MAPK1 (mitogen-activated protein kinase 1) exons 2, 3 and 4. MAPK1 is a signaling molecule of the MAPK signaling pathway that plays important roles in regulating cell proliferation, differentiation, invasion and migration [42]. However, the role of hsa_circ_0004872 in disease is unknown. In this study, we found that the expression of hsa_circ_0004872 was dramatically decreased in GC tissues compared with adjacent nontumor tissues. Consistent with previous reports, hsa_circ_0004872 was more stable than the linear RNA and not easily degraded by RNase R. The biological function assays in vitro showed that hsa_circ_0004872 inhibited GC cells proliferation, invasion and migration. Moreover, in vivo nude mice experiments also verified the tumor-suppressor activity of hsa_circ_0004872. In addition, Zhu et al. [43] also found that hsa_circ_0004872 was downregulated in glioblastoma, revealing the tumor suppressor role of hsa_circ_0004872 in different tumors.

It is well known that exonic circRNAs are usually located in the cytoplasm. Our FISH experiment also showed that hsa_circ_0004872 mainly existed in the cytoplasm. The most frequently reported function of exonic circRNAs is their role as an “miRNA sponge” to bind with miRNAs and inhibit the function of the miRNAs, thus protecting the target genes from miRNA-mediated degradation [19]. For example, a report from Han et al. showed that exonic circMTO1 suppressed hepatocellular carcinoma progression by acting as a sponge of oncogenic miR-9 and increasing p21 expression [10]. A study from Wang et al. showed that circNT5E acted as a miR-422a sponge to regulate the proliferation, apoptosis, invasion and migration of glioblastoma cells [16]. In this study, we confirmed the “miRNA sponge” role of hsa_circ_0004872 by an RNA binding protein immunoprecipitation experiment (RIP), in which we used an antibody against Ago2, an important component of the miRNA-induced silencing complex [44], to immunoprecipitate Ago2-miRNA-circRNA complexes and then amplified hsa_circ_0004872 from the complexes. Then, we used different databases to predict the potential miRNAs and selected potential oncogenic miRNAs to investigate their interaction with hsa_circ_0004872 by biotin-coupled probe pull-down assay. Our results showed that hsa_circ_0004872 was able to bind with miR-224. Furthermore, FISH and dual luciferase reporter assays confirmed the colocalization and interaction between hsa_circ_0004872 and miR-224 in GC cells. Further statistical analysis of the expression of hsa_circ_0004872 and miR-224 in GC tissues showed a negative correlation between them.

We next sought to elucidate the biological role and mechanism of miR-224 in GC cells and found that miR-224 exerted critical functions in GC progression and metastasis by targeting p21 and Smad4. p21 is an inhibitor of the cell cycle and affects the progression of the cell cycle, leading to the inhibition of cell proliferation [34]. Smad4 is a member of the TGF-β signaling pathway and plays an important role in cell growth, apoptosis, invasion and migration [30]. To further investigate whether the biological function of hsa_circ_0004872 in GC cells was associated with miR-224, we performed a rescue experiment. Our findings suggested that hsa_circ_0004872 influences the biological functions of GC by regulating p21 and Smad4 via miR-224.

It has been reported that the formation of circRNAs is regulated by the regulatory proteins MBl [22], QKI [36, 37], adenosine deaminase 1 (ADAR1) [38, 39] and other RNA binding proteins. To determine which factors may affect the level of hsa_circ_0004872 in GC, we analyzed the public database NCBI GEO (GSE27342 and GSE66229) and found only ADAR1 was elevated in GC tissues compared with adjacent nontumor tissues in the both GEO databases. ADAR1, a member of the ADAR family, has two subtypes p110 and p150 [45, 46]. ADAR1 catalyzes the deamination of adenosine and converts adenosine (A) to inosine (I). ADAR1 was reported to strongly inhibit the production of circRNA, and its A-to-I editing process usually occurs near the reverse complementary matches (RCMs) region, a structure believed to be critical for circRNA formation [41]. Shi et al. reported that ADAR1 in hepatocellular carcinoma suppresses the expression of CircARSP91 [41]. Therefore, we investigated the regulatory effect of ADAR1 on hsa_circ_0004872 expression and confirmed that ADAR1 could decrease the level of hsa_circ_0004872 in GC cells. Taken together, our findings demonstrated that the overexpression of ADAR1 contributed to the downregulation of hsa_circ_0004872 in GC and that the low expression of hsa_circ_0004872 promoted the tumorigenesis and progression of GC by regulating p21 and Smad4 via miR-224.

We further investigated whether Smad4, as a transcription factor, was able to regulate ADAR1 expression, thereby forming a regulatory feedback loop of Smad4/ADAR1/hsa_circ_0004872/miR-224/Smad4. We used the Jaspar database to predict the binding sites of Smad4 in the promoter region of ADAR1 and found five potential sites. As expected, our results showed that Smad4 overexpression decreased the expression level of ADAR1 and increased the level of hsa_circ_0004872 in GC cells. ChIP and dual luciferase reporter experiments verified that Smad4 could bind to the promoter region of ADAR1 to regulate its expression in GC cells. Furthermore, according to the public database NCBI GEO (GSE66229), the expression level of Smad4 was significantly low in GC tissues. Statistical analysis showed that there was a remarkable negative correlation between ADAR1 and Smad4 in GC tissues. The Kaplan-Meier Plotter Database indicated that higher expression of ADAR1 or lower expression of Smad4 in GC tissues resulted in a shorter survival period.

In summary, in this study, we investigated the expression, role and regulatory mechanism of the tumor-suppressor circular RNA hsa_circ_0004872 in GC. We found that hsa_circ_0004872 was dramatically downregulated in GC tissues, which was due at least partially to the overexpression of ADAR1. hsa_circ_0004872 inhibited the proliferation, invasion and migration of GC cells by acting as a “miRNA sponge” to bind with miR-224 and increase the expression of the endogenous miR-224 targets p21 and Smad4. Moreover, Smad4, as a transcription factor, could also regulate hsa_circ_0004872 expression by directly binding to the promoter region of ADAR1 and decreasing its expression. Therefore, we revealed a novel regulatory feedback loop formed by hsa_circ_0004872/miR-224/Smad4/ADAR1 in GC. Our findings may provide new ideas and targets for the diagnosis and treatment of GC.

## Supplementary information

**Additional file 1: Figure S1.** qRT-PCR analysis of the expression of hsa_circ_0004872, hsa_circ_0002483,hsa_circ_0000847, hsa_circ_0001566 in 42 paired GC tissues and corresponding nontumor tissues.

**Additional file 2: Figure S2.** qRT-PCR analysis of the expression of hsa_circ_0004872 in BGC-823 and SGC-7901 cells transfected with hsa_circ_0004872 overexpression vector (pLCDH-circ_4872) (A) or the miR-224 binding site mutated hsa_circ_0004872 overexpression vector ((pLCDH-circ_4872-Mut) (B).

**Additional file 3: Figure S3.** hsa_circ_0004872 siRNAs promote the proliferation, invasion and migration of GC cells. (A) Schematic representation of the siRNA sequences specifically target the junction site of hsa_circ_0004872. (B) qRT-PCR analysis of hsa_circ_0004872 and MAPK1 mRNA level in BGC-823 cells transfected with hsa_circ_0004872 siRNAs (si-circ_4872) or the control siRNA. (C) EdU analysis of the cell proliferation ability in BGC-823 cells transfected with the si-circ_4872 or the control siRNA.Representative images are shown. Scale bar: 20 μm. (D) Statistical analysis of the EdU-positive cell ratio in the cells transfected with si-circ_4872 or the control siRNA. (E) The scratch wound healing assays in BGC-823 cells transfected with the si-circ_4872 or the control siRNA. Scale bar: 500 μm. (F) Statistical analysis of the cell migration in the scratch wound healing assays.The data are expressed as the means±SD from three experiments. (G) Transwell invasion and migration assay in BGC-823 cells transfected with the si-hsa_circ_0004872 or the control siRNA. Scale bar: 100 μm. (H) Statistical analysis of the cell numbers passing through the transwell chamber in the transfected BGC-823 cells. The data are expressed as the means±SD from three experiments.

**Additional file 4: Figure S4.** qRT-PCR analysis of the expression of hsa_circ_0004872 in different GC cells.

**Additional file 5: Figure S5.** Schematic diagam of dual luciferase vector. (A) Schematic diagam of dual luciferase vector pMIRGLO-circ_4872-WT/Mut. Upper: diagram of the luciferase reporter construct containing the sequences of hsa_circ_0004872. The mutations were generated at the predicted miR-224 binding sites in the hsa_circ_0004872 sequences. Lower: the predicted complementary sequences of miR-224 in the sequences of hsa_circ_0004872. (B) Schematic diagam of dual luciferase vector pMIR-Smad4(p21)-WT/Mut. Upper: diagram of the luciferase reporter construct containing 3’UTR sequences of Smad4 (p21). The mutations were generated at the predicted miR-224 binding sites located in the 3’UTR of Smad4(p21). Lower: the predicted complementary sequences of miR-224 in the 3’UTR of Smad4 (p21). (C) Schematic diagram of dual luciferase vector pGL3-ADAR1-WT/Mut. Upper: diagram of the luciferase reporter construct containing promoter sequence of ADAR1. The mutations were generated at the predicted Smad4 binding sites located in promoter sequence of ADAR1. Lower: the predicted complementary sequences of Smad4 in promoter sequence of ADAR1.

**Additional file 6: Figure S6.** qRT-PCR analysis of the expression of miR-224 (A) and ADAR1 (B) in 39 paired GC tissues and corresponding nontumor tissues.

**Additional file 7: Figure S7.** miR-224 inhibitor inhibited the proliferation, invasion and migration in GC cells (A) The expression level of miR-224 was analyzed with qRT-PCR in BGC-823 and SGC-7901 cells transfected with miR-224 inhibitor or the control inhibitor. (B) EdU analysis of the cell proliferation ability in BGC-823 and SGC-7901 cells transfected with miR-224 inhibitor or the control inhibitor. Scale bar: 20 μm. (C) Statistical analysis of the EdU-positive cell ratio in the transfected cells. (D) CCK-8 analysis of the cell proliferation ability in BGC-823 and SGC-7901 cells transfected with miR-224 inhibitor or the control inhibitor. (E) The scratch wound healing assays of the migration ability in transfected BGC-823 and SGC-7901 cells. Scale bar: 500 μm. (F) Statistical analysis of the scratch wound healing assays. (G) Transwell assay of the migration (without matrigel) and invasion ability (with matrigel) in BGC-823 and SGC-7901 cells transfected with miR-224 inhibitor or the control inhibitor. Scale bar: 100 μm. (H) Statistical analysis of the cell numbers passing through the transwell chamber in the transfected BGC-823 and SGC-7901 cells. All datas were the means ± SD.

**Additional file 8: Figure S8.** The expression of ADAR1, MBl and QKI were analyzed in NCBI GEO database GSE27342 and GSE66229. (A) The expression level of ADAR1 was analyzed with paired t-tests (*n* = 80, *p* < 0.0001) in GEO database GSE27342. (B) The expression level of ADAR1 was analyzed with unpaired t-tests (*n* = 400, *p* < 0.0001) in GSE66229. (C) The expression level of MBL was analyzed with paired t-tests (*n* = 80, *p* = 0.7912) in GSE27342. (D) The expression level of MBL was analyzed with unpaired t-tests (*n* = 400, *p* = 0.7566) in GSE66229. (E) The expression level of QKI was analyzed with paired t-tests (*n* = 80, *p* = 0.3101) in GSE27342. (F) The expression level of QKI was analyzed with unpaired t-tests (*n* = 400, *p* < 0.0001) in GSE66229.

**Additional file 9: Table S1.** Patients, tumor characteristics and hsa_circ_0004872 expression in GC samples.

**Additional file 10: Table S2.** siRNA sequences in the research.

**Additional file 11: Table S3.** Primer sequences in this study.

**Additional file 12: Table S4.** Probe sequences of hsa_circ_0004872 and miR-224 in this study.

**Additional file 13: Table S5.** CircRNA-seq analysis of the differentially expressed circRNAs in GC tissue and corresponding nontumor tissue.

**Additional file 14: Table S6**. Predicted miRNAs with potential binding ability with hsa_circ_0004872 in different databases.

## Data Availability

All data generated or analyzed during this study are included either in this article or in the supplementary files.

## Abbreviations

3′-UTR: 3′- untranslated region
CCK-8: Cell counting kit- 8
EdU: 5-Ethynyl-2′-deoxyuridine
circRNA: Circular RNA
FISH: Fluorescence in situ hybridization
GC: Gastric cancer
gDNA: Genomic DNA
HE: Hematoxylin and eosine
miRNA: MicroRNA
mRNA: Messenger ribonucleic acid
qRT-PCR: Real-time quantification PCR
RIP: RNA-binding protein immunoprecipitation
RT-PCR: Reverse transcriptase PCR
siRNA: Small interfering RNA
ChIP: Chromatin immunoprecipitation
