# Supplementary figures and images for: Circular RNA hsa_circ_0004872 inhibits gastric cancer progression via the miR-224/Smad4/ADAR1 successive regulatory circuit
Source: Mol Cancer. 2020 Nov 10;19:157. doi: 10.1186/s12943-020-01268-5 (PMC7654041; doi:10.1186/s12943-020-01268-5)

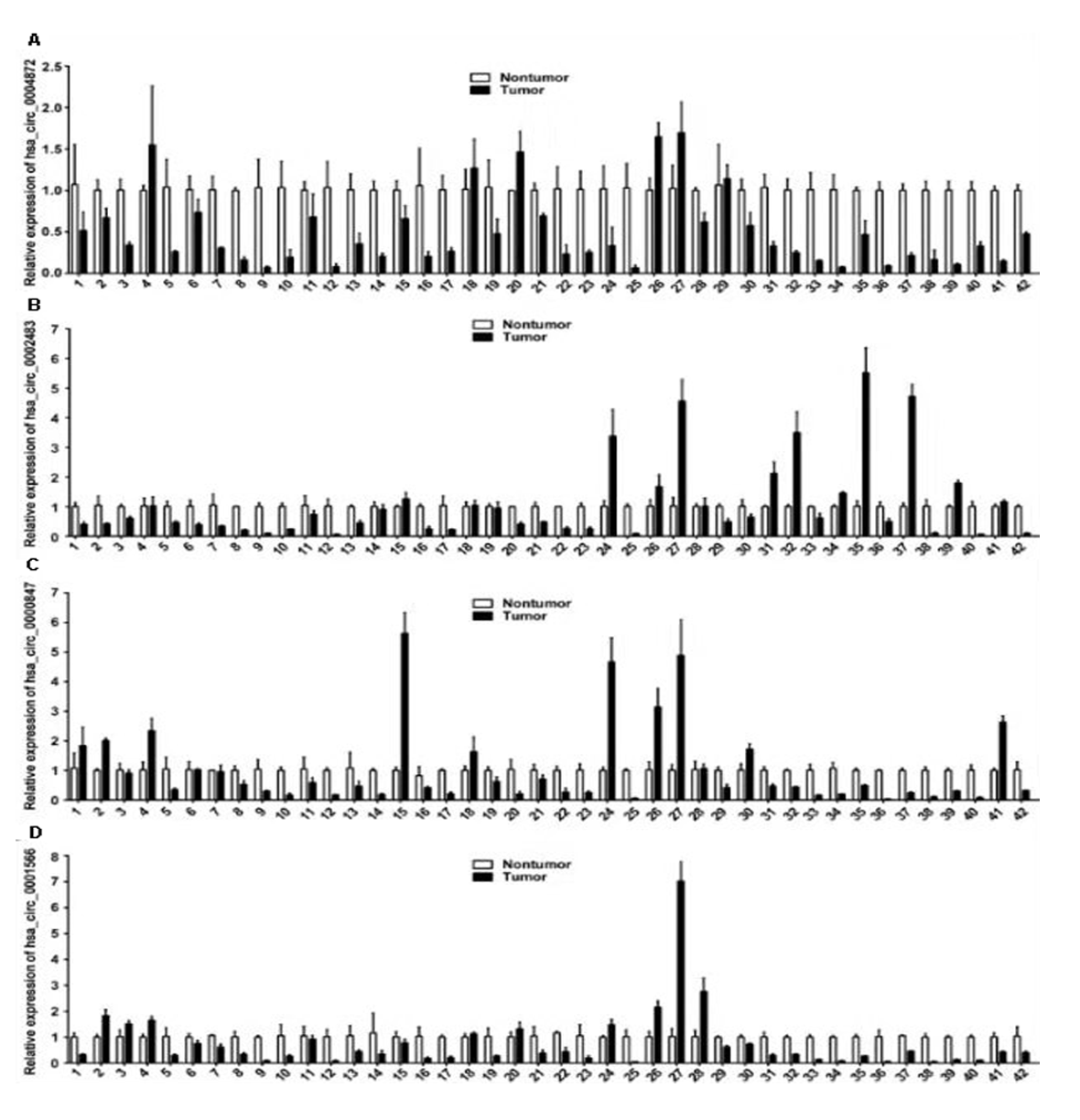

Supplement: Supplementary file 1 — Additional file 1: Figure S1. qRT-PCR analysis of the expression of hsa_circ_0004872, hsa_circ_0002483,hsa_circ_0000847, hsa_circ_0001566 in 42 paired GC tissues and corresponding nontumor tissues. [file 12943_2020_1268_MOESM1_ESM.tif]

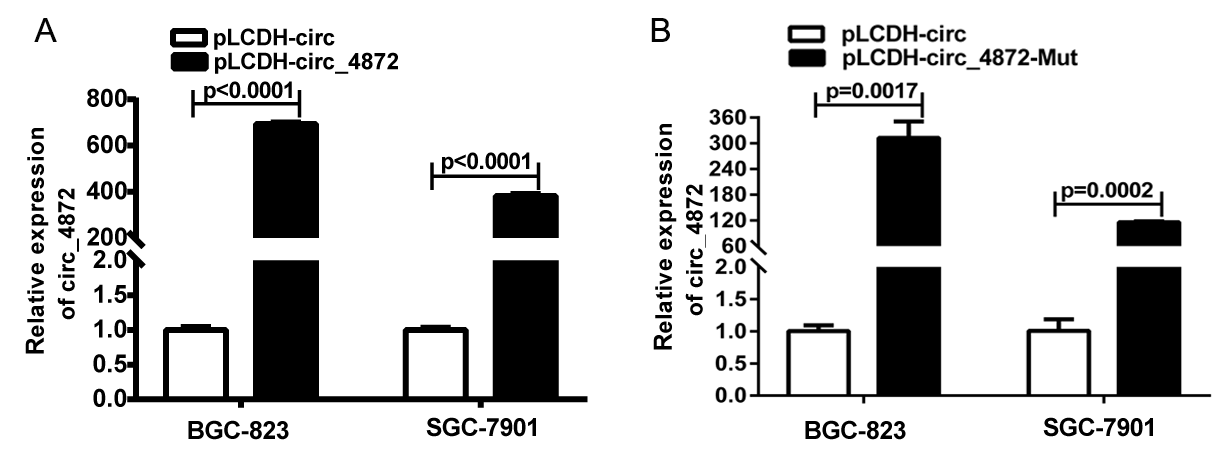

Supplement: Supplementary file 2 — Additional file 2: Figure S2. qRT-PCR analysis of the expression of hsa_circ_0004872 in BGC-823 and SGC-7901 cells transfected with hsa_circ_0004872 overexpression vector (pLCDH-circ_4872) (A) or the miR-224 binding site mutated hsa_circ_0004872 overexpression vector ((pLCDH-circ_4872-Mut) (B). [file 12943_2020_1268_MOESM2_ESM.tif]

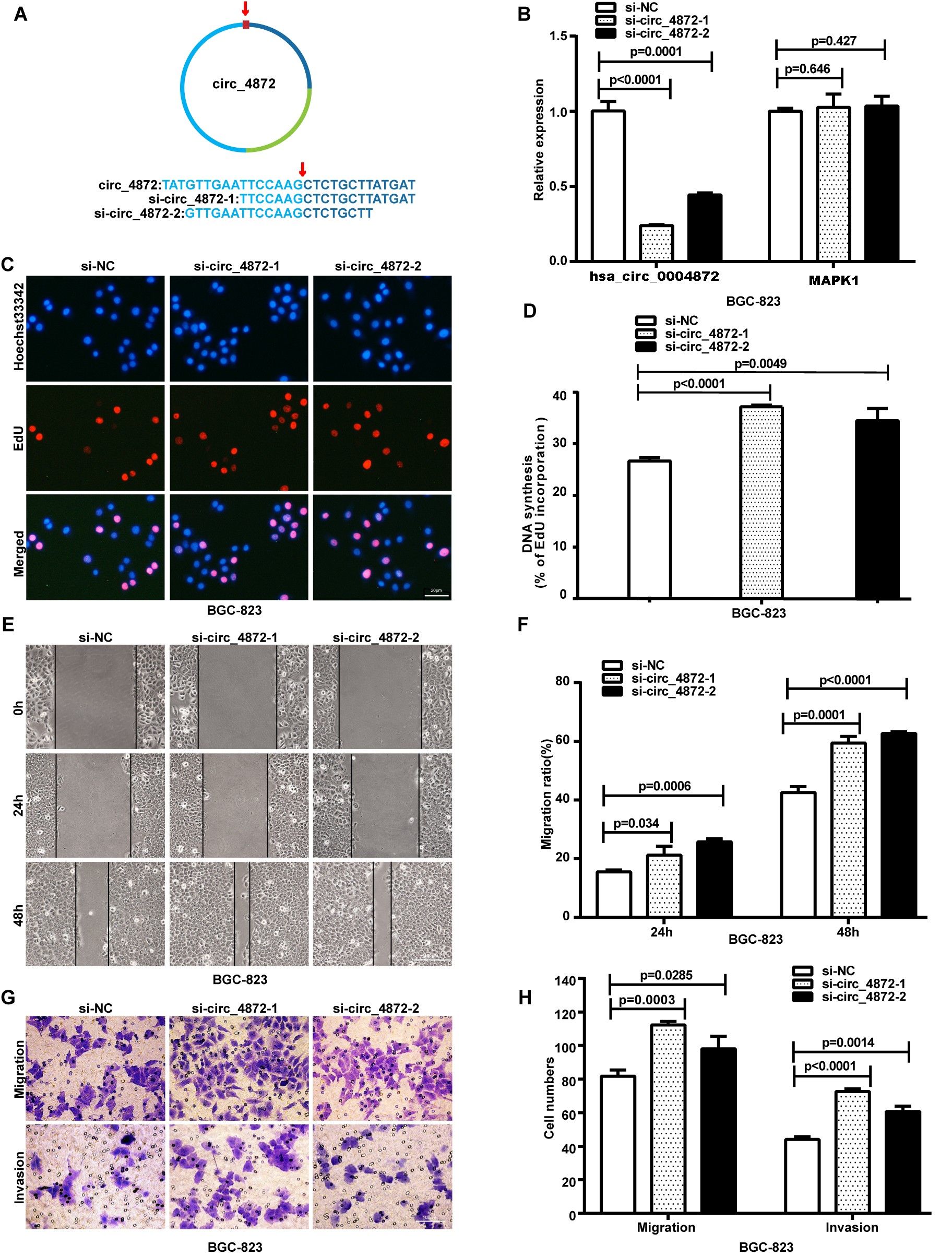

Supplement: Supplementary file 3 — Additional file 3: Figure S3. hsa_circ_0004872 siRNAs promote the proliferation, invasion and migration of GC cells. (A) Schematic representation of the siRNA sequences specifically target the junction site of hsa_circ_0004872. (B) qRT-PCR analysis of hsa_circ_0004872 and MAPK1 mRNA level in BGC-823 cells transfected with hsa_circ_0004872 siRNAs (si-circ_4872) or the control siRNA. (C) EdU analysis of the cell proliferation ability in BGC-823 cells transfected with the si-circ_4872 or the control siRNA.Representative images are shown. Scale bar: 20 μm. (D) Statistical analysis of the EdU-positive cell ratio in the cells transfected with si-circ_4872 or the control siRNA. (E) The scratch wound healing assays in BGC-823 cells transfected with the si-circ_4872 or the control siRNA. Scale bar: 500 μm. (F) Statistical analysis of the cell migration in the scratch wound healing assays.The data are expressed as the means±SD from three experiments. (G) Transwell invasion and migration assay in BGC-823 cells transfected with the si-hsa_circ_0004872 or the control siRNA. Scale bar: 100 μm. (H) Statistical analysis of the cell numbers passing through the transwell chamber in the transfected BGC-823 cells. The data are expressed as the means±SD from three experiments. [file 12943_2020_1268_MOESM3_ESM.tif]

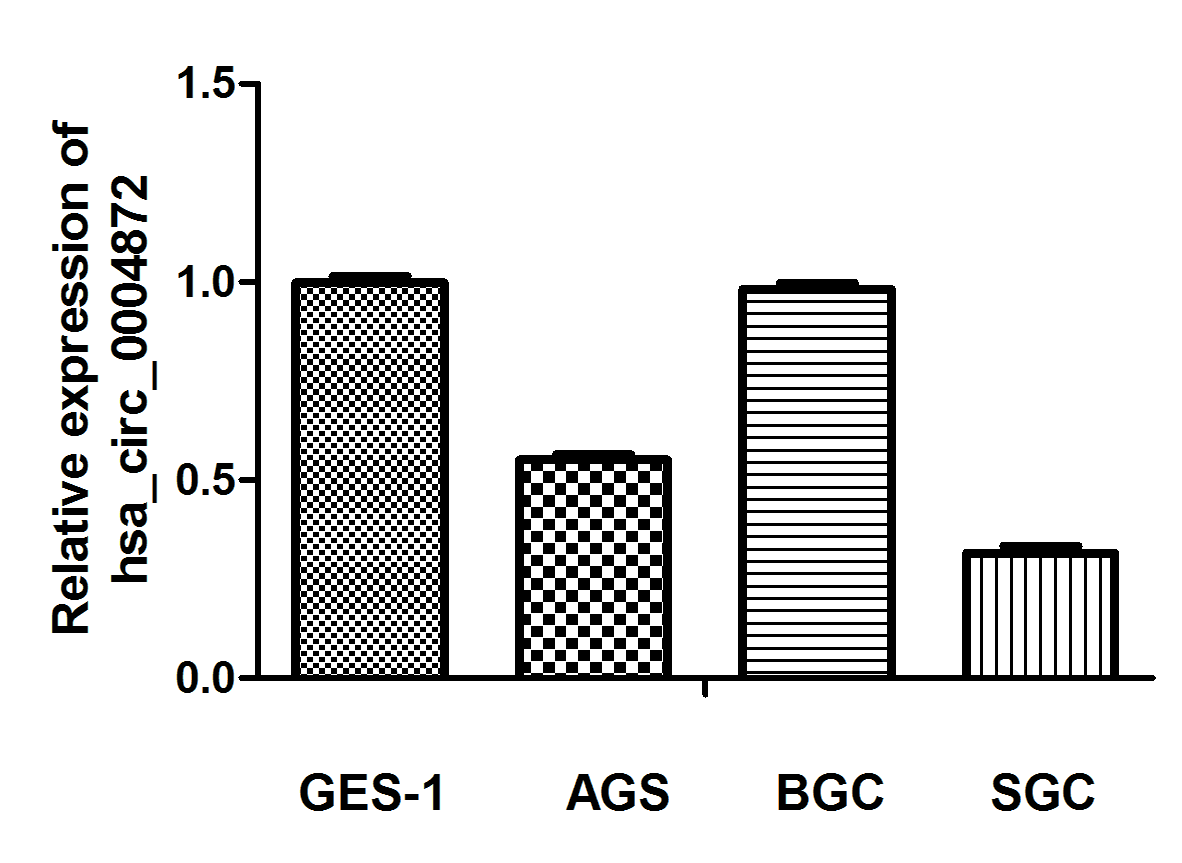

Supplement: Supplementary file 4 — Additional file 4: Figure S4. qRT-PCR analysis of the expression of hsa_circ_0004872 in different GC cells. [file 12943_2020_1268_MOESM4_ESM.tif]

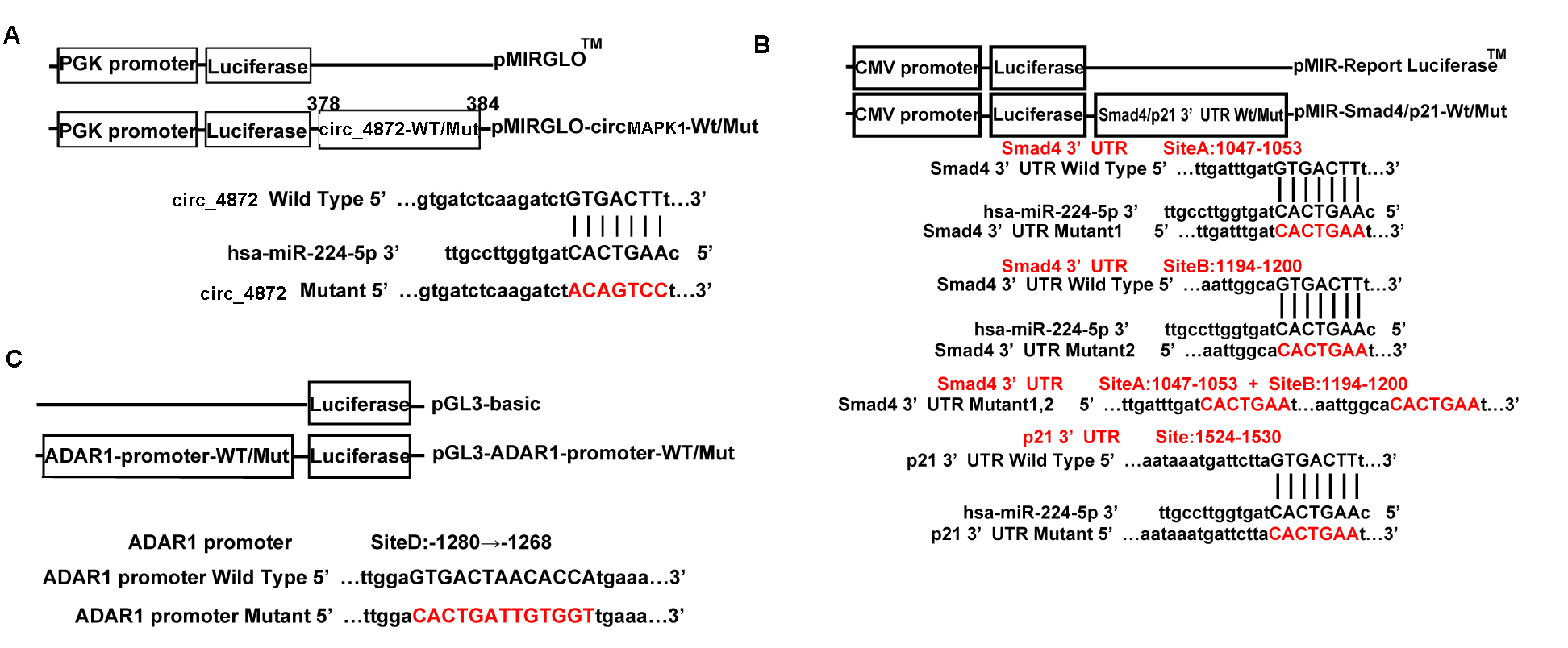

Supplement: Supplementary file 5 — Additional file 5: Figure S5. Schematic diagam of dual luciferase vector. (A) Schematic diagam of dual luciferase vector pMIRGLO-circ_4872-WT/Mut. Upper: diagram of the luciferase reporter construct containing the sequences of hsa_circ_0004872. The mutations were generated at the predicted miR-224 binding sites in the hsa_circ_0004872 sequences. Lower: the predicted complementary sequences of miR-224 in the sequences of hsa_circ_0004872. (B) Schematic diagam of dual luciferase vector pMIR-Smad4(p21)-WT/Mut. Upper: diagram of the luciferase reporter construct containing 3’UTR sequences of Smad4 (p21). The mutations were generated at the predicted miR-224 binding sites located in the 3’UTR of Smad4(p21). Lower: the predicted complementary sequences of miR-224 in the 3’UTR of Smad4 (p21). (C) Schematic diagram of dual luciferase vector pGL3-ADAR1-WT/Mut. Upper: diagram of the luciferase reporter construct containing promoter sequence of ADAR1. The mutations were generated at the predicted Smad4 binding sites located in promoter sequence of ADAR1. Lower: the predicted complementary sequences of Smad4 in promoter sequence of ADAR1. [file 12943_2020_1268_MOESM5_ESM.tif]

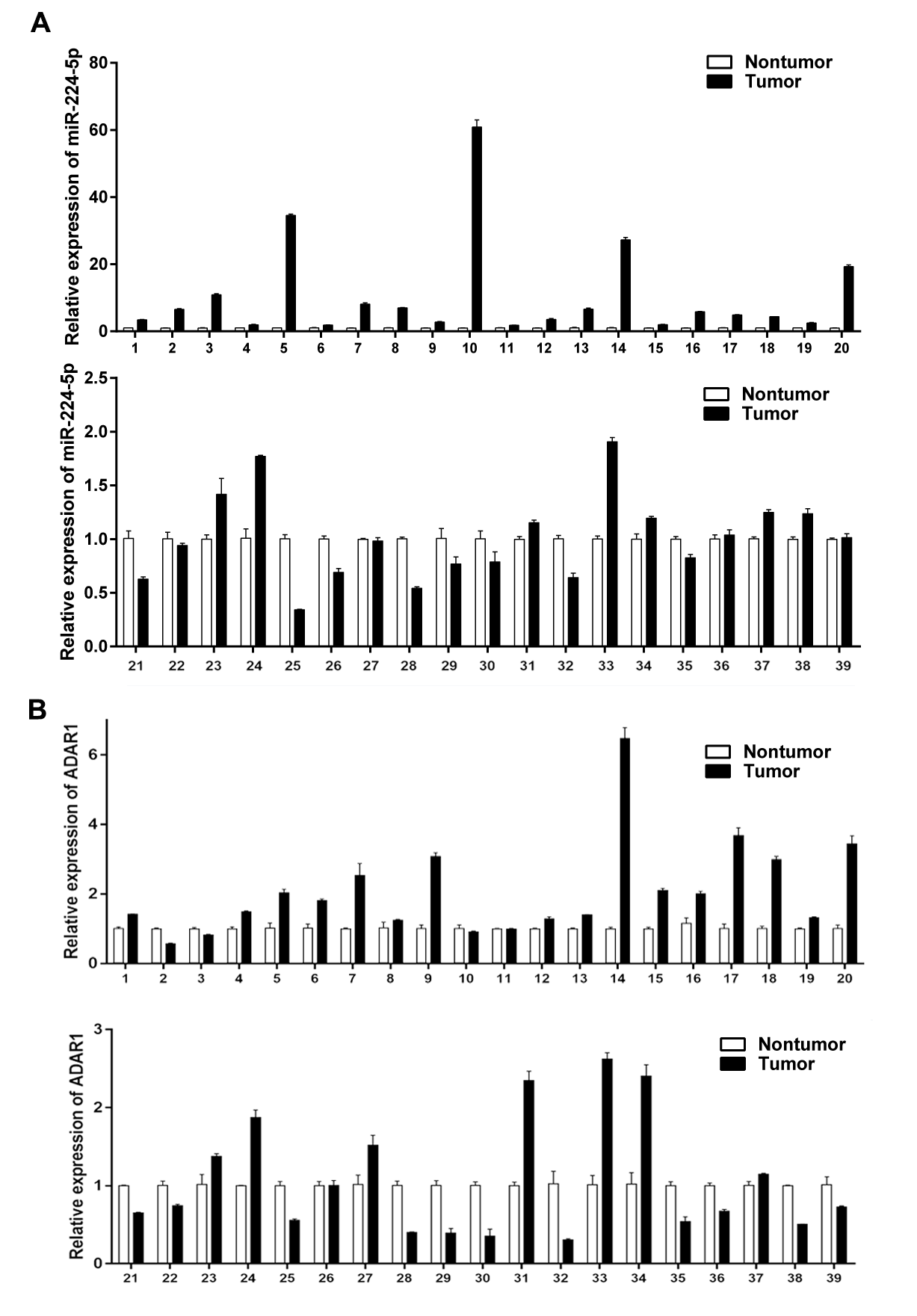

Supplement: Supplementary file 6 — Additional file 6: Figure S6. qRT-PCR analysis of the expression of miR-224 (A) and ADAR1 (B) in 39 paired GC tissues and corresponding nontumor tissues. [file 12943_2020_1268_MOESM6_ESM.tif]

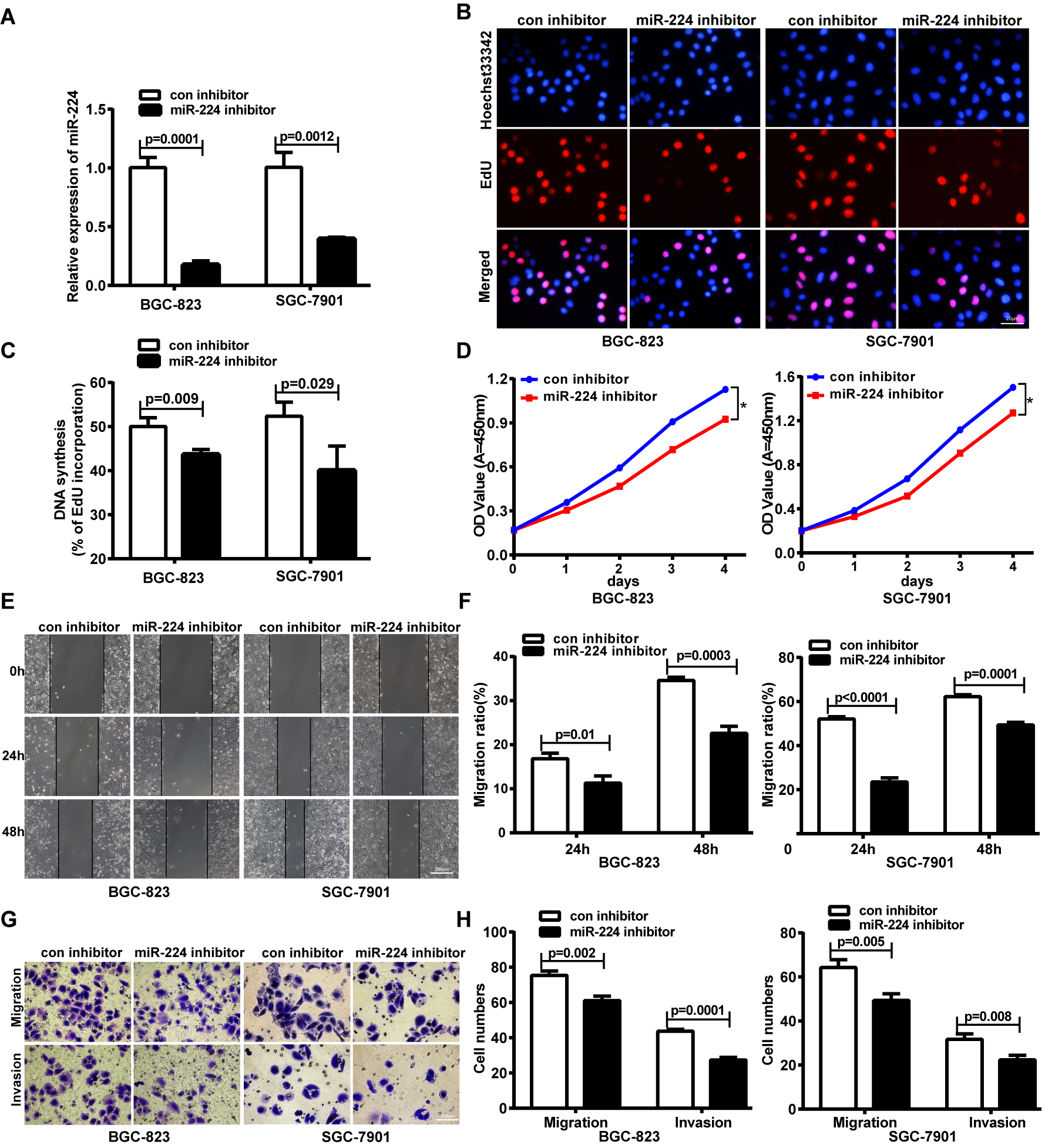

Supplement: Supplementary file 7 — Additional file 7: Figure S7. miR-224 inhibitor inhibited the proliferation, invasion and migration in GC cells (A) The expression level of miR-224 was analyzed with qRT-PCR in BGC-823 and SGC-7901 cells transfected with miR-224 inhibitor or the control inhibitor. (B) EdU analysis of the cell proliferation ability in BGC-823 and SGC-7901 cells transfected with miR-224 inhibitor or the control inhibitor. Scale bar: 20 μm. (C) Statistical analysis of the EdU-positive cell ratio in the transfected cells. (D) CCK-8 analysis of the cell proliferation ability in BGC-823 and SGC-7901 cells transfected with miR-224 inhibitor or the control inhibitor. (E) The scratch wound healing assays of the migration ability in transfected BGC-823 and SGC-7901 cells. Scale bar: 500 μm. (F) Statistical analysis of the scratch wound healing assays. (G) Transwell assay of the migration (without matrigel) and invasion ability (with matrigel) in BGC-823 and SGC-7901 cells transfected with miR-224 inhibitor or the control inhibitor. Scale bar: 100 μm. (H) Statistical analysis of the cell numbers passing through the transwell chamber in the transfected BGC-823 and SGC-7901 cells. All datas were the means ± SD. [file 12943_2020_1268_MOESM7_ESM.tif]

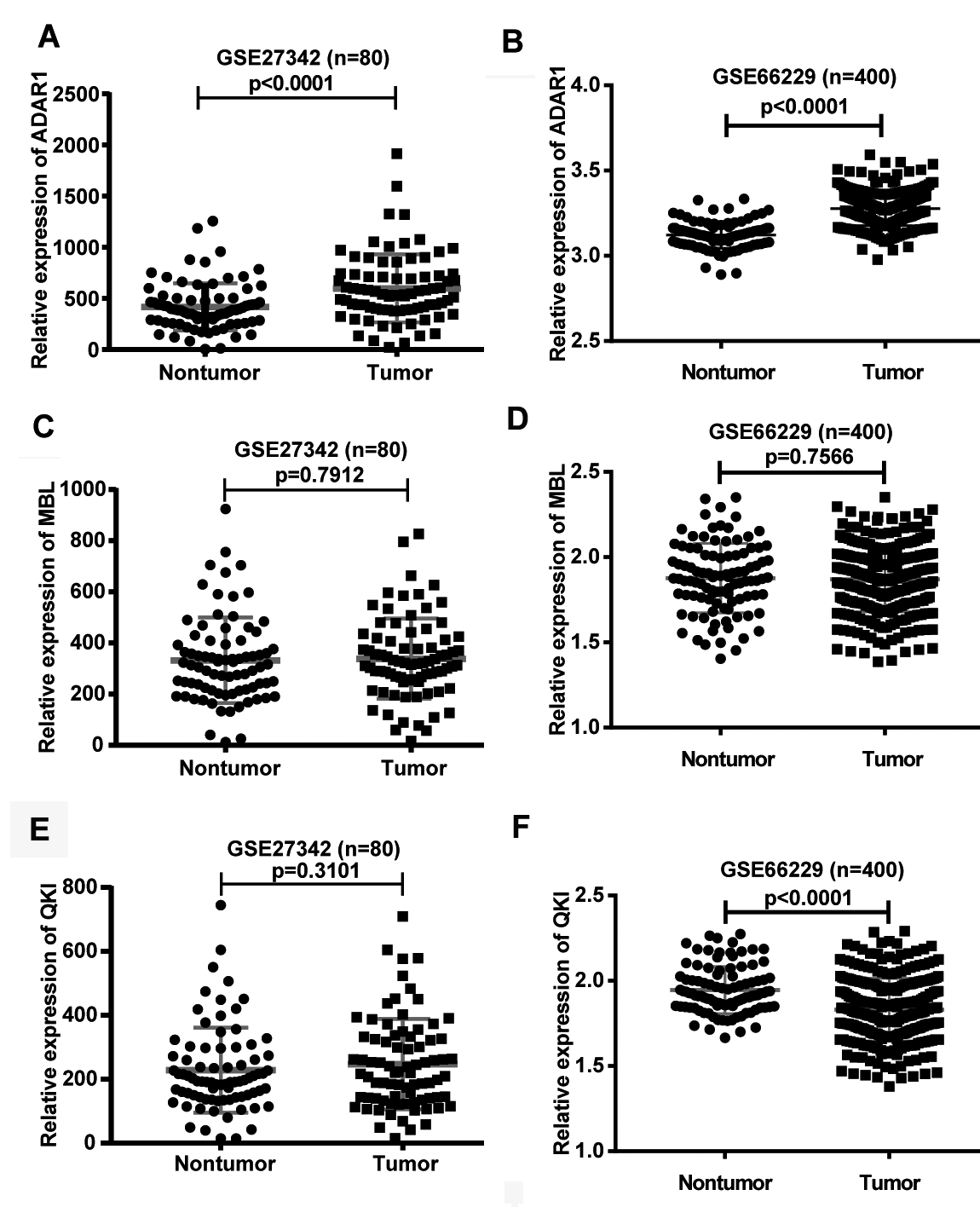

Supplement: Supplementary file 8 — Additional file 8: Figure S8. The expression of ADAR1, MBl and QKI were analyzed in NCBI GEO database GSE27342 and GSE66229. (A) The expression level of ADAR1 was analyzed with paired t-tests (n = 80, p < 0.0001) in GEO database GSE27342. (B) The expression level of ADAR1 was analyzed with unpaired t-tests (n = 400, p < 0.0001) in GSE66229. (C) The expression level of MBL was analyzed with paired t-tests (n = 80, p = 0.7912) in GSE27342. (D) The expression level of MBL was analyzed with unpaired t-tests (n = 400, p = 0.7566) in GSE66229. (E) The expression level of QKI was analyzed with paired t-tests (n = 80, p = 0.3101) in GSE27342. (F) The expression level of QKI was analyzed with unpaired t-tests (n = 400, p < 0.0001) in GSE66229. [file 12943_2020_1268_MOESM8_ESM.tif]
