# Supplementary material for: Circular RNA hsa_circ_0004872 inhibits gastric cancer progression via the miR-224/Smad4/ADAR1 successive regulatory circuit
Source: Mol Cancer. 2020 Nov 10;19:157. doi: 10.1186/s12943-020-01268-5 (PMC7654041; doi:10.1186/s12943-020-01268-5)
Supplement: Supplementary file 9 — Additional file 9: Table S1. Patients, tumor characteristics and hsa_circ_0004872 expression in GC samples. [file 12943_2020_1268_MOESM9_ESM.docx]

Table S1. Patients and tumor characteristics, hsa_circ_0004872 expression in GC samples

| **No.** | **Gender** | **Age（yr）** | **TNM** | **Size（diameter,cm）** | **Relative Expression level（ΔCt*）** |
| --- | --- | --- | --- | --- | --- |
| 1 | M | 68 | T3N3bM0 | 6 | 10.03 |
| 2 | F | 52 | T4aN3bM1 | 4.5 | 12.37 |
| 3 | M | 69 | T4aN0M0 | 7 | 11.16 |
| 4 | F | 46 | T4aN1M0 | 7 | 12.30 |
| 5 | M | 58 | T4aN1M0 | 2 | 12.13 |
| 6 | M | 68 | T4aN2M0 | 5.5 | 12.68 |
| 7 | F | 59 | T3N3bM1 | 5.5 | 10.39 |
| 8 | M | 55 | T4aN3bM0 | 7.5 | 10.21 |
| 9 | M | 57 | T1bN0M0 | 1 | 12.64 |
| 10 | F | 69 | T3N1M0 | 3 | 10.42 |
| 11 | M | 45 | T4aN3aM1 | 10 | 12.32 |
| 12 | F | 65 | T4aN3aM0 | 12 | 11.27 |
| 13 | M | 51 | T3N2M0 | 3 | 11.28 |
| 14 | M | 52 | T4aN2M0 | 10.5 | 10.82 |
| 15 | F | 69 | T4aN3bM0 | 14 | 9.43 |
| 16 | M | 75 | T3N3aM0 | 6 | 10.01 |
| 17 | M | 73 | T4aN3bM0 | 7 | 8.80 |
| 18 | F | 49 | T4aN1M0 | 3 | 8.34 |
| 19 | F | 77 | T2N0M0 | 1 | 9.56 |
| 20 | M | 52 | T2N1M0 | 2 | 10.81 |
| 21 | F | 37 | T2N0M0 | 1 | 6.51 |
| 22 | F | 66 | T3N1M0 | 8 | 9.74 |
| 23 | F | 40 | T3N3bM0 | 6 | 11.50 |
| 24 | F | 30 | T3N0M0 | 2.2 | 9.59 |
| 25 | M | 80 | T4aN1M0 | 8 | 12.05 |
| 26 | F | 56 | T1bN0M0 | 3 | 11.82 |
| 27 | F | 57 | T3N3aM0 | 5.5 | 10.86 |
| 28 | M | 64 | T3N2M1 | 5 | 9.62 |
| 29 | M | 74 | T2N1M0 | 2 | 9.75 |
| 30 | M | 66 | T4N3M0 | 9 | 12.80 |
| 31 | F | 69 | T3N3aM0 | 4 | 10.96 |
| 32 | M | 70 | T2N0M0 | 9 | 11.82 |
| 33 | M | 73 | T4aN3aM0 | 6 | 10.51 |
| 34 | F | 68 | T4aN2M0 | 6 | 12.23 |
| 35 | F | 66 | T4aN3aM0 | 3 | 11.98 |
| 36 | M | 68 | T4N3M1 | 7 | 13.07 |
| 37 | M | 65 | T3N0M0 | 7 | 12.05 |
| 38 | F | 56 | T2N0M0 | 6 | 12.69 |
| 39 | M | 54 | T4aN3aM0 | 7 | 11.15 |
| 40 | F | 60 | T3N3aM0 | 2.5 | 11.78 |
| 41 | M | 62 | T4N2M0 | 6 | 12.23 |
| 42 | M | 72 | T4N3M0 | 2.5 | 11.27 |
| 43 | M | 52 | T4N3M0 | 6.5 | 13.52 |
| 44 | M | 80 | T4N3M1 | 11 | 11.313 |
| 45 | M | 60 | T4N3M0 | 5.5 | 11.83 |
| 46 | M | 59 | T4N3M0 | 6.5 | 10.89 |
| 47 | F | 37 | T4aN3aM1 | 13 | 10.79 |
| 48 | F | 56 | T4N2M0 | 5.4 | 10.88 |
| 49 | M | 54 | T2N0M0 | 4 | 5.903 |
| 50 | F | 64 | T4aN2M0 | 3.5 | 8.92 |
| 51 | F | 52 | T4aN1M0 | 9 | 11.66 |
| 52 | M | 66 | T4aN3M0 | 9 | 9.20 |
| 53 | M | 61 | T4aN0M0 | 5 | 9.40 |
| 54 | M | 45 | T4N2M0 | 10 | 11.84 |
| 55 | M | 37 | T4N3M0 | 6 | 13.62 |
| 56 | M | 63 | T4aN3aM0 | 5.5 | 10.99 |
| 57 | M | 75 | T1bN0M0 | 1 | 8.71 |
| 58 | M | 56 | T4bN1M0 | 5.5 | 12.49 |
| 59 | F | 69 | T4aN1M0 | 4.5 | 7.40 |
| 60 | M | 49 | T4aN0M0 | 3 | 7.06 |
| 61 | M | 64 | T1N0M0 | 1 | 8.27 |
| 62 | M | 50 | T2N0M0 | 4 | 8.72 |
| 63 | F | 38 | T4N1M0 | 8 | 9.68 |
| 64 | M | 62 | T1N1M0 | 4 | 9.45 |
| 65 | M | 69 | T1N1M0 | 5 | 8.32 |
| 66 | F | 43 | T1N0M0 | 3 | 5.52 |
| 67 | M | 70 | T4N3M1 | 8.5 | 10.22 |
| 68 | M | 73 | T4N2M0 | 6 | 10.05 |
| 69 | F | 66 | T4N2M0 | 3.5 | 8.42 |
| 70 | M | 69 | T4N3M1 | 7.5 | 10.55 |
| 71 | M | 50 | T4aN3M0 | 4 | 8.46 |
| 72 | F | 52 | T4N2M0 | 9 | 10.26 |
| 73 | M | 68 | T4N3M1 | 7 | 13.12 |
| 74 | M | 74 | T2N0M0 | 6 | 8.88 |
| 75 | M | 79 | T4N2M0 | 5.5 | 10.98 |
| 76 | M | 59 | T4N3M0 | 5 | 8.19 |

*ΔCt=the ct value of hsa_circ_0004872-the ct value of internal control （β_2_-M）

The higher ΔCt value implies lower expression.
