# Supplementary material for: Circular RNA hsa_circ_0004872 inhibits gastric cancer progression via the miR-224/Smad4/ADAR1 successive regulatory circuit
Source: Mol Cancer. 2020 Nov 10;19:157. doi: 10.1186/s12943-020-01268-5 (PMC7654041; doi:10.1186/s12943-020-01268-5)
Supplement: Supplementary file 10 — Additional file 10: Table S2. siRNA sequences in the research. [file 12943_2020_1268_MOESM10_ESM.docx]

**Table S2. siRNA sequences in the research**

| **Name** | **SiRNA Sequence** |
| --- | --- |
| hsa_circ_0004872 siRNA1 | TTCCAAGCTCTGCTTATGATT |
| hsa_circ_0004872 siRNA2 | GTTGAATTCCAAGCTCTGCTT |
| ADAR1 siRNA | GCATCTGACCCGTGCTATTdTdT |
| Negative control siRNA | CCUACAUCCCGAUCGAUGAUGUUGA |
