# Supplementary material for: Circular RNA hsa_circ_0004872 inhibits gastric cancer progression via the miR-224/Smad4/ADAR1 successive regulatory circuit
Source: Mol Cancer. 2020 Nov 10;19:157. doi: 10.1186/s12943-020-01268-5 (PMC7654041; doi:10.1186/s12943-020-01268-5)
Supplement: Supplementary file 11 — Additional file 11: Table S3. Primer sequences in this study. [file 12943_2020_1268_MOESM11_ESM.docx]

**Table S3. Primer sequences in this study**

| **Name** | **Primer Sequence** |
| --- | --- |
| hsa_circ_0004872 WT luciferase reporter vector (293bp) | F:5’-CCGCTCGAGTATAGTACAGGACCTCATGG-3’ |
|  | R:5’-GCGTCGACACATAATTTCTGGAGCCCTG-3’ |
| hsa_circ_0004872 Mut luciferase reporter vector | F1:5’-CACCTGTGATCTCAAGATCTACAGTCCCGGCCTGGC  CCGTGTTGCAGA-3’  R:5’-GCGTCGACACATAATTTCTGGAGCCCTG-3’ |
|  | F:5’-CCGCTCGAGTATAGTACAGGACCTCATGG-3’  R1:5’-TCTGCAACACGGGCCAGGCCGGGACTGTAGATCTT  GAGATCACAGGTG-3’ |
| Smad4 WT 3'UTR luciferase reporter vector(606bp) | F:5’-GGACTAGTCTAGGCACAAGGTTGGTTGC-3’ |
|  | R:5’-CCCAAGCTTCATCACTGAGATTGGACTCA-3’ |
| Smad4 3'UTR luciferase reporter vector (Mut1) | F1:5’-CTTGATTTGATCACTGAATTTTTGGTATA-3’  R:5’-CCCAAGCTTCATCACTGAGATTGGACTCA-3’ |
|  | F:5’-GGACTAGTCTAGGCACAAGGTTGGTTGC-3’  R1:5’-TATACCAAAAATTCAGTGATCAAATCAAG-3’ |
| Smad4 3'UTR luciferase reporter vector (Mut2) | F2:5’-CAATTGGCACACTGAATGTATAGAG-3’  R:5’-CCCAAGCTTCATCACTGAGATTGGACTCA-3’ |
|  | F:5’-GGACTAGTCTAGGCACAAGGTTGGTTGC-3’  R2:5’-CTCTATACATTCAGTGTGCCAATTG-3’ |
| p21 WT 3'UTR luciferase reporter vector(229bp) | F:5’-GGACTAGTACTTGGAGTATTGGGGTCTG-3’ |
|  | R:5’-CCCAAGCTTCAAGTAAAGTCACTAAGAATC-3’ |
| p21 3'UTR luciferase reporter vector (Mut) | F:5’-GGACTAGTACTTGGAGTATTGGGGTCTG-3’  R1:5’-CCCAAGCTTCTTACAAGTATTCAGTGTAAGAATC-3’ |
| ADAR1 WT promoter reporter vector(2011bp) | F:5’-CGACGCGTTGGCTCCGGTTCAATTTCGC-3’ |
|  | R:5’-CCCAAGCTTTGCATCCTTGCTGCCACCTGGT-3’ |
| ADAR1Mut promoter reporter vector | F1:5’-GTTAATTTGGACACTGATTGTGGTTGAAAGGGCAT  CAGCTGGAGATACTG-3’  R:5’-CCCAAGCTTTGCATCCTTGCTGCCACCTGGT-3’ |
|  | F:5’-CGACGCGTTGGCTCCGGTTCAATTTCGC-3’  R1:5’-CAGTATCTCCAGCTGATGCCCTTTCAACCACAATC  AGTGTCCAAATTAAC-3’ |
| β2-M (Convergent)  (250bp) | F:5’-GAATTGCTATGTGTCTGGGT-3’ |
|  | R:5’-CATCTTCAAACCTCCATGATG-3’ |
| β2-M (Divergent)  (120bp) | F:5’-AGATGAGTATGCCTGCCGTG-3’ |
|  | R:5’-TCATCCAATCCAAATGCGGC-3’ |
| Hsa_circ_0004872 (Divergent primer,181bp) | F:5’-GTTGCAGATCCAGACCATGA-3’ |
|  | R:5’-CAGGGTTCTCTGGCAGTAGG-3’ |
| Hsa_circ_0004872 (Convergent primer,180bp) | F:5’-ACAACACCTCAGCAATGACCA-3’ |
|  | R:5’-TGGTCTGGATCTGCAACACG-3’ |
| β-actin  (101bp) | F:5’-TTGCCGACAGGATGCAGAA-3’  R:5’-GCCGATCCACACGGAGTACT-3’ |
| p21  (112bp) | F:5’-CTGGAGACTCTCAGGGTCGAA-3’  R:5’-AGGCTTCCTGTGGGCGGATT-3’ |
| Smad4  (108bp) | F:5’-GCTGCTGGAATTGGTGTTGATG-3’ |
|  | R:5’-AGGTGTTTCTTTGATGCTCTGTCT-3’ |
| ADAR1  (169bp) | F:5’-AAGTCCTGCAGCGACCGTGC-3’ |
|  | R:5’-TCTCCCCGAGCCGAATGCCA-3’ |
| ADAR1-ChIP-SiteAB(-1993to -1981, -1959 to -1947) (156bp) | F:5’-CTTGCTGCCACCTGGTCTAA-3’ |
|  | R:5’-CGAAAGACAACTTCACAGGAGA-3’ |
| ADAR1-ChIP-SiteC  (-1590 to -1578) (118bp) | F:5’-AGCAGACTAGCTAAAGGATGGC-3’ |
|  | R:5’-AAACGCCCCATTCGATCATT-3’ |
| ADAR1-ChIP-SiteD  (-1280 to -1268) (176bp) | F:5’-GGGGAAGGCCTCCCTTGTA-3’ |
|  | R:5’-AGTATCTCCAGCTGATGCCCT-3’ |
| ADAR1-ChIP-SiteE  (-760 to -748) (141bp) | F:5’-TCCTAAATTGTTCACTGCTGCTG-3’ |
|  | R:5’-GGAGCGCAGATCTCGTCAAAC-3’ |
