# Supplementary material for: Circular RNA hsa_circ_0004872 inhibits gastric cancer progression via the miR-224/Smad4/ADAR1 successive regulatory circuit
Source: Mol Cancer. 2020 Nov 10;19:157. doi: 10.1186/s12943-020-01268-5 (PMC7654041; doi:10.1186/s12943-020-01268-5)
Supplement: Supplementary file 12 — Additional file 12: Table S4. Probe sequences of hsa_circ_0004872 and miR-224 in this study. [file 12943_2020_1268_MOESM12_ESM.docx]

**Table S4. Probe sequences of hsa_circ_0004872 and miR-224 in this study.**

| **Name** | **Probe Sequences** |
| --- | --- |
| hsa_circ_0004872-CY3 for FISH | ATAAGCAGAGCTTGGAATT |
| miR-224-FITC for FISH | CTAAACGGAACCACTAGTGACTTGA |
| hsa_circ_0004872 probe for RNA pull-down | AAGCAGAGCTTGGAATTCAA-bio |
| NC probe for RNA pull-down | TAGTATACCAAGGAGAGTAC-bio |
| hsa_circ_0004872 probe for Northern blot | Dig-TGACATTATCATAAGCAGAGCTTGGAATTCAACATAATTT |
| U6 probe for Northern blot | Dig-TATCGTTCCAATTTTAGTATATGTGCTGCCGAAGCGAG |
