# Supplementary material for: Circular RNA hsa_circ_0004872 inhibits gastric cancer progression via the miR-224/Smad4/ADAR1 successive regulatory circuit
Source: Mol Cancer. 2020 Nov 10;19:157. doi: 10.1186/s12943-020-01268-5 (PMC7654041; doi:10.1186/s12943-020-01268-5)
Supplement: Supplementary file 14 — Additional file 14: Table S6. Predicted miRNAs with potential binding ability with hsa_circ_0004872 in different databases. [file 12943_2020_1268_MOESM14_ESM.docx]

**Table S6** **Predicted miRNAs with potential binding ability with hsa_circ_0004872 in different databases**

| **Database** | **Predicted miRNA** |
| --- | --- |
| circular RNA interactome, BIOINF, starBase | hsa-miR-224, hsa-miR-513a-5p,  hsa-miR-599, hsa-miR-654-3p |
| circular RNA interactome, BIOINF | hsa-miR-224, hsa-miR-513a-5p, hsa-miR-599,  hsa-miR-654-3p, hsa-miR-657, hsa-miR-1233-3p,  hsa-miR-1299, hsa-miR-146b-3p, hsa-miR-602,  hsa-miR-487a-5p |
| BIOINF, starBase | hsa-miR-224, hsa-miR-513a-5p, hsa-miR-599,  hsa-miR-654-3p, hsa-miR-10a-5p, hsa-miR-761,  hsa-miR-10b-5p, hsa-miR-15a-5p, hsa-miR-15b-5p,  hsa-miR-16-5p, hsa-miR-195-5p, hsa-miR-214-3p,  hsa-miR-2681-3p, hsa-miR-335-5p, hsa-miR-588,  hsa-miR-3619-5p, hsa-miR-370-5p, hsa-miR-424-5p, hsa-miR-486-5p, hsa-miR-497-5p, hsa-miR-516b-5p, hsa-miR-552-3p, hsa-miR-552-5p, hsa-miR-579-3p,  hsa-miR-4726-5p, hsa-miR-6838-5p, hsa-miR-616-3p, hsa-miR-664b-3p, hsa-miR-4640-5p, hsa-miR-4701-5p, |
